# smoothclust: Spatial smoothing and boundary-density summaries for spatial transcriptomics

**DOI:** 10.64898/2026.10.26.761912

**Published:** 2026-10-30

**Authors:** Lauren Rusnak, Lukas M. Weber

**Affiliations:** 1 Department of Biostatistics, Boston University School of Public Health, Boston, MA, USA

**Keywords:** spatial transcriptomics, spatial domains, spatial clustering, tissue architecture, boundary density, sample-level summaries, Bioconductor

## Abstract

Spatial transcriptomics enables the measurement of gene expression while preserving tissue organization. A common analysis task is the identification of spatial domains, defined here as spatially coherent regions with consistent expression profiles or cell-type mixtures and represented using categorical spatial tissue maps. However, while numerous methods have been developed to identify spatial domains, interpretable summary measures to quantify spatial domain organization and tissue architecture at the sample level remain less standardized. Here, we present smoothclust, a framework for spatial domain analysis consisting of two components: a spatial-smoothing-based clustering workflow for generating spatial domain labels, and a boundary density (BD) metric that provides local and sample-level summaries of label transitions on a spatial graph. Building on established categorical spatial statistics, the raw BD metric is calculated from the proportion of neighboring spatial locations that are assigned to different domain labels, and requires only spatial coordinates and domain labels as inputs. The metric can be calculated using either our spatial-smoothing-based domain labels or labels from external clustering methods or manual annotations. Relative BD is a composition-adjusted summary, calculated by dividing raw BD by its expectation under random relabeling with the observed label counts. By quantifying label transitions, our framework translates spatial domain label maps into interpretable local and sample-level summary measures of tissue architecture, facilitating exploratory analyses and comparisons across samples and biological conditions. We demonstrate the approach through evaluations across datasets from several spatial transcriptomics platforms and simulations. The spatial-smoothing workflow and BD metric are implemented in the smoothclust R package, which is freely available from Bioconductor and GitHub.

## 1 Introduction

Spatial transcriptomics technologies measure high-dimensional gene expression together with spatial coordinates in intact tissue sections, enabling direct investigation of tissue organization in cancer, neuroscience, immunology, development, and other settings [1, 2, 3]. Technological platforms include sequencing-based and imaging-based approaches, and differ in spatial resolution, transcriptome coverage, sensitivity, and whether measurements correspond to spots, bins, segmented cells, or subcellular transcript detections [2, 4]. Computational analyses of these data consist of complex workflows integrating a number of preprocessing and downstream analyses [5, 6, 7, 8]. A key analysis task is the identification of spatial domains, defined here as spatially coherent regions with consistent expression profiles or cell-type mixtures, which are represented using categorical spatial tissue maps. Depending on the biological context, these maps may delineate anatomical regions, tumor, stromal, or immune compartments, cell-type niches, or other regions of shared molecular composition. Spatial domains may be identified algorithmically using spatially-aware clustering algorithms, or may be defined based on manual annotations, histology-derived segments, or anatomical regions. Spatial domain maps provide an interpretable intermediate representation between individual spatial locations and whole tissue sections, supporting analyses of domain composition, within-domain expression, domain-level differential expression, relationships to histology, and interfaces between tissue compartments.

Numerous computational methods have been developed for spatially-aware clustering and spatial domain identification, applying various methodological approaches. Representative approaches include Bayesian modeling of spatial neighborhoods and multi-sample clustering, spatially-aware dimension reduction and factor analysis, graph convolutional or graph attention models, spatial representation learning and integration, histology or neighborhood features, and expression smoothing followed by clustering [9, 10, 11, 12, 13, 14, 15, 16, 17, 18]. These methods differ in the modalities of information they use, the scale and strength of spatial regularization, the type and granularity of labels they produce, and whether they are designed for individual sections or multiple samples. Benchmarking studies have emphasized that performance depends on tissue structure, technological platform, spatial resolution, available annotations, sample-integration strategy, and the downstream scientific question, with no single method performing uniformly best across settings [19, 20].

Multi-sample spatial transcriptomics studies introduce an additional layer of challenges for analysis by creating a need to make results comparable across tissue sections, individuals, or biological conditions [20]. One strategy is to analyze multiple sections jointly by integrating expression spaces, aligning tissue sections, or fitting models that directly accept multiple sections as input. This includes general single-cell integration approaches such as Harmony [21] and others, as well as spatially-aware methods for alignment, integration, and multi-sample domain detection [22, 10, 12, 15, 23, 18]. These approaches can borrow information across samples, reduce the need for post hoc matching of section-specific labels, and support comparisons in a common embedding or label space. However, they also require choices or assumptions about batch correction, correspondence of tissue structures and labels, spatial alignment, comparable resolution, and whether sample-specific biological variation should be preserved or removed.

A complementary strategy is to calculate sample-level summary measures after categorical labels have been obtained, whether from independent single-section analyses, an integrated multi-sample workflow, histology-derived segmentation, published computational annotations, or manual annotations. When informative summary measures are available, this approach can provide a simpler and interpretable route for exploratory visualization, comparisons across biological groups or conditions, or association analyses with clinical covariates. While spatial domain detection has received substantial methodological attention, the development and interpretation of summary measures for quantifying tissue architecture at the sample level remain less standardized. Most domain detection workflows return a spatial map of labels, which is visually informative but difficult to compare systematically across large cohorts or experimental conditions. Summary-based analyses can complement joint clustering and integration without necessarily requiring alignment of individual spatial locations across sections.

Existing spatial analysis frameworks provide several approaches to this problem. These include multi-view sample representations in scFeatures [24], cell-type colocalization, neighborhood enrichment, and spatial interaction analyses in frameworks such as Squidpy, SPIAT, and spicyR [6, 25, 26], multiscale diversity summaries in MESA [27], and tissue representations based on persistent local relationship patterns in Kasumi [28]. HTA provides an entropy-based sample-level heterogeneity summary by averaging local heterogeneity across spatial regions [29]. CellCharter additionally characterizes spatial cluster components using shape-related measures [30]. These approaches describe different aspects of tissue architecture, and the choice of summary depends on the scientific question; no single spatial summary fully establishes clustering accuracy or biological validity [31, 32, 33, 34].

Here, we develop a spatial domain boundary density (BD) metric as a simple label-source-agnostic measure of label-transition frequency within a tissue section. Given spatial coordinates and a domain label for each spatial location, the raw BD metric quantifies the proportion of local nearest-neighbor relationships that connect locations assigned to different labels. Intuitively, at each spatial location, this represents the proportion of neighboring spatial locations that are assigned to different domain labels. This quantity is a normalized discordant join count and connects directly to classical categorical spatial statistics and graph homophily [35, 36, 37]. Spatial autocorrelation, pair-specific joins, and local indicators of spatial association provide related descriptions of spatial structure [38, 39, 40], with relevant implementations including stJoincount [41] and Voyager [42].

Related recent approaches include SHouT [43], inter-label connectivity in Spatialproteomics [44], a spatial-connectivity criterion used for benchmarking by [45], neighborhood entropy analyses provided by ClustSIGNAL [18], and stEDGE [46]. SHouT reports global edge homophily, defined as the fraction of spatial-graph edges connecting cells of the same type, and global normalized entropy of the sample-wide label composition as sample-level scores [43]. On an identical spatial graph, sample-level raw BD is the complement of global homophily, although the default graph definitions and intended label maps differ. At the local level, SHouT’s egophily measures agreement between a focal cell and cells in its neighborhood, making it closely related to the complement of local BD. Spatialproteomics [44] defines inter-label connectivity as the fraction of a cell’s neighbors assigned to a different cell-type label, and its average across cells for sample comparisons; with matching labels and spatial neighborhoods excluding the focal cell, the local measure and equally-weighted average are equivalent to local and sample-level raw BD. [45] use a spatial-connectivity criterion for benchmarking niche identification that averages the fraction of neighboring cells assigned to the same domain label as the focal cell; under matching labels, spatial neighborhoods, and averaging conventions, this is equivalent to one minus sample-level raw BD. ClustSIGNAL calculates cell-specific Shannon entropy from the initial-subcluster composition of each neighborhood and uses these values to control adaptive expression smoothing; the resulting neighborhood entropy distributions can also be summarized at the sample level [18]. These entropy summaries quantify neighborhood diversity, whereas local BD quantifies disagreement between a focal location’s label and the labels of its neighbors. stEDGE uses a consensus-based boundary signal to guide spatial domain reconstruction and hierarchical organization [46]. With matching neighborhoods and exclusion of self-relations, its initial boundary signal can be expressed as local raw BD averaged over an ensemble of labelings. However, stEDGE uses this signal to reconstruct domains, whereas we use BD to summarize existing label maps.

An optional composition adjustment to the raw BD metric produces relative BD, calculated by dividing raw BD by its expectation under random relabeling with the observed label counts. This expectation can be calculated analytically from the label counts. Relative BD expresses observed transition frequency relative to a section-specific reference, complementing the direct summary provided by raw BD. It also has a connection to Geary’s C [39], connecting BD to established spatial statistics: for binary labels and matching spatial weights, relative BD equals Geary’s C; for multiple labels, it is a variance-weighted average of Geary’s C calculated for the corresponding label-indicator variables. The adjustment accounts for label composition through the random-label expectation but does not make relative BD independent of composition, label definitions, or spatial scale. Comparisons therefore require comparable labels, spatial graphs, and sampling units.

We present smoothclust, a framework for spatial domain analysis consisting of two components: a spatial-smoothing-based clustering workflow for generating spatial domain labels, and a BD metric that provides local and sample-level summaries of label transitions on a spatial graph. The BD metric requires only spatial coordinates and categorical labels as inputs, and can be applied to labels from smoothclust, external clustering methods, published annotations, or manual annotations. Low raw BD indicates relatively few local label transitions, while high raw BD indicates frequent local transitions, which may reflect dense interfaces between tissue compartments, intermixed biological structures, fragmented domains, or noisy label assignments. Our contribution combines an accessible R/Bioconductor implementation with analyses of datasets from several spatial transcriptomics technologies demonstrating how BD summaries depend on label definitions, spatial scale, and the datasets being studied. By quantifying spatial domain label transitions, our framework translates categorical spatial maps into interpretable local and sample-level summary measures of tissue architecture, facilitating exploratory analyses and sample-level comparisons.

## 2 Results

### 2.1 BD metric summarizes GBM/glioma tissue organization

We first applied smoothclust independently to 19 Greenwald et al. GBM/glioma Visium sections [47] (Supplementary Table S1) and compared the resulting labels with a matched non-spatial k-means baseline (Figure 1). Both workflows used the same retained spots and input genes, normalization and transformation procedures, and downstream analysis settings. Variable genes and principal components were estimated separately from smoothed and unsmoothed expression. This comparison therefore evaluated the effect of including smoothing in the complete clustering workflow.

Local raw BD highlighted interfaces between domain labels for smoothclust (Figure 1A–B), while the matched non-spatial baseline showed more frequent label transitions (Figure 1C). In section zh1019t1 at K=6, sample-level raw BD was 0.130 for smoothclust and 0.425 for the baseline. Across all 19 sections and K=3,…,8, the baseline had higher raw BD in all 114 matched comparisons (Figure 1D–E). At K=6, mean raw BD was 0.091 for smoothclust and 0.386 for the baseline. Sample-level raw BD values for smoothclust across sections and cluster numbers are summarized in Figure 1F. These results demonstrate the reduction in label transitions after spatial smoothing.

Lower raw BD values after smoothing were also observed with kernel and k-nearest-neighbor smoothing (Supplementary Figure S1). Increasing the smoothing scale generally lowered raw BD (Supplementary Figure S2). In contrast, holding labels fixed and increasing the BD neighborhood size broadened local interface signals and generally raised the sample-level value (Supplementary Figure S3). These analyses illustrate the distinct effects of smoothing scale and the neighborhood used to summarize the resulting labels.

### 2.2 Simulations illustrate the composition adjustment and over-smoothing

Composition simulations on a 30 × 30 grid illustrated the distinction between raw and relative BD (Supplementary Figure S4). Random arrangements at 10:90, 30:70, and 50:50 label compositions had different raw BD expectations, while relative BD was centered near 1, with means ranging from 1.000 to 1.002 across the three compositions. With counts fixed at 450:450, relative BD increased from 0.034 for two coherent halves to 0.391 and 0.836 after exchanging labels at 10% and 30% of locations, respectively, 0.976 for a random arrangement, and 1.931 for a checkerboard. At this fixed composition, raw and relative BD differ only by a constant scaling.

Straight-interface maps with 10:90, 30:70, and 50:50 compositions further illustrated the role of the random-label expectation. With the graph, interface orientation, and interface extent held fixed, raw BD was approximately 0.017 in all three maps, while relative BD was 0.096, 0.040, and 0.034, respectively. Thus, similar observed transition frequencies can yield different relative BD values because the expected frequencies differ. The adjustment therefore does not make relative BD independent of composition in spatially structured maps.

An additional over-smoothing simulation illustrated why lower BD does not necessarily indicate better domain recovery (Supplementary Figure S5). For two broad domains and a small island, recovery was highest at a uniform-smoothing bandwidth of 4% of the coordinate range (ARI 0.973; island F1 0.872). At 30%, ARI fell to 0.663 and island F1 to 0.105 even though raw and relative BD were lower. This simulation illustrates how excessive smoothing can erase a small domain while further reducing label transitions; the best-performing bandwidth is specific to this example.

### 2.3 BD metric summarizes external spatial clustering labels or manual annotations

We next applied BD to domain labels that we generated using three external spatial clustering algorithms. Across the 19 sections for the GBM/glioma Visium dataset, median sample-level raw BD was 0.103 for spatial clustering labels generated using BayesSpace [9], 0.085 for BANKSY [17], and 0.155 for ClustSIGNAL [18], compared with 0.071 for smoothclust clustering and 0.343 for the non-spatial baseline (Supplementary Figure S6). BayesSpace, BANKSY, smoothclust, and the baseline used K=5, while ClustSIGNAL used its default labeling settings and returned a variable number of labels [18]. For section zh1019t1, the domain labels generated by BayesSpace, BANKSY, and ClustSIGNAL are shown in Figure 2A–C, with the corresponding local raw BD maps highlighting label transitions in Figure 2D–F. These analyses illustrate the flexibility of BD as a summary measure that can be calculated from any source of categorical spatial domain labels.

We also applied BD directly to four sets of published annotation labels from Greenwald et al. [47], illustrating the use of BD with existing label maps rather than labels generated by independent clustering. Across the 13 sections with these annotations, coarse organization-zone labels had lower transition frequencies than dominant-metaprogram labels and deposited layer annotations (Supplementary Figure S7). Median relative BD was 0.117 for coarse organization zones, 0.162 for malignancy-refined zones, 0.771 for dominant metaprograms, and 0.746 for layer labels. Metaprogram and layer relative BD were negatively associated with the published structured-zone fraction (Spearman ρ=-0.90 and −0.86, respectively). Since the organization zones were derived from local coherence of the metaprogram assignments, these associations show agreement between related descriptions of tissue organization.

We also examined regional variation within individual tumors by comparing metaprogram composition and relative BD across sections from the same tumor. Across 12 regional sections from four tumors, we observed substantial within-tumor differences, particularly for ZH881 and ZH916 (Supplementary Figure S8).

### 2.4 BD metric provides a sample-level summary of myocardial organization

We next applied BD to a different biological setting to illustrate its use for descriptive comparisons of tissue organization across sample categories. We calculated BD using the nine published cell-type-niche labels available from the myocardial-infarction Visium dataset of Kuppe et al. [48], across 28 tissue sections from 20 patients. Median relative BD was 0.923 in myogenic-enriched, 0.656 in ischaemic-enriched, and 0.737 in fibrotic-enriched sections (Figure 3A). All relative BD values were below 1, indicating fewer label transitions than expected under random relabeling with the observed counts in each section, as illustrated by the comparison of observed and expected raw BD (Figure 3B). Local raw BD maps showed the transitions contributing to representative sample-level values (Supplementary Figure S9). Higher relative BD in myogenic-enriched sections indicates more frequent transitions among the published cell-type-niche labels relative to their section-specific composition references. This pattern may reflect interspersion of compositionally distinct niches within broadly myogenic tissue, whereas some injured sections may contain larger patches dominated by particular niche labels.

Two ischaemic sections were dominated by a single niche, occupying 99.0% and 87.8% of spots (red points in Figure 3A and open circles in Figure 3B; composition profiles in Supplementary Figure S10). Excluding these sections changed the ischaemic median relative BD only slightly, from 0.656 to 0.652. This illustrates how BD can provide section-level summaries even for sections with strongly imbalanced label compositions. Comparisons across the author-defined tissue categories were descriptive and section-weighted, with repeated patient identities indicated in Figure 3A.

To assess sensitivity to neighborhood size, we recalculated relative BD using k=18 and k=36 while holding the published niche labels fixed. Section-level rankings remained strongly correlated with those at k=6 (Spearman ρ=0.989 and 0.984, respectively; Supplementary Figure S11), with median absolute changes of 0.025 and 0.039. Category medians retained the ordering myogenic-enriched > fibrotic-enriched > ischaemic-enriched.

### 2.5 BD metric provides annotation-specific cortical summaries

To further investigate the effects of annotation granularity on BD, we analyzed a human frontal-cortex Visium dataset from Miyoshi et al. [49]. The dataset includes published annotations generated with BayesSpace [9] that define nine cortical and white-matter regions across 39 tissue sections. Using these labels, we obtained relative BD values ranging from 0.127 to 0.312, with a median of 0.198 (Supplementary Figure S12). Plotting section-level values alongside label proportions provides a joint view of spatial organization and composition. Complete annotation and local raw BD maps are shown in Supplementary Figure S13.

For the original nine-region annotation, we also assessed sensitivity to BD neighborhood size. Relative BD rankings at k=18 and k=36 remained strongly correlated with those at k=6 (Spearman ρ=0.965 and 0.925, respectively), although values shifted substantially (median absolute changes of 0.078 and 0.145, respectively; Supplementary Figure S11).

To investigate the effects of annotation granularity, we then collapsed WM1–WM3 into a single white-matter label. The original and merged annotations, together with their corresponding local raw BD maps for a representative section, are shown in Figure 4A–B. Across all 39 sections, raw BD decreased in 38 and was unchanged in one, with a median paired change (merged minus original) of −0.0254 (Figure 4C). Merging labels also decreased or left unchanged the random-label expectation (Figure 4D), since pairs assigned to different white-matter labels become concordant when at least two of the merged labels are present. Median relative BD decreased to 0.175, and section rankings changed (Spearman ρ=0.591; Figure 4E). Relative BD decreased in 37 sections, was unchanged in one, and increased in one, with a median paired change of −0.019. Since relative BD is the ratio of observed to expected BD, it can increase when the expectation decreases proportionally more than the observed value. These results illustrate how label granularity affects both the magnitude and ordering of sample-level summaries.

To examine BD summaries using manual anatomical annotations, we also analyzed a human dorsolateral prefrontal cortex (DLPFC) Visium dataset from Maynard et al. [50]. Using the published cortical-layer and white-matter annotations for 12 sections from three donors, relative BD ranged from 0.0666 to 0.1180. The median absolute difference between six pairs of directly adjacent sections was 0.00125, with a maximum of 0.00601 (Supplementary Figure S14). These observations provide a descriptive assessment of agreement in section-level relative BD between adjacent sections. Complete manual annotation and corresponding local raw BD maps are shown in Supplementary Figure S14.

### 2.6 High-resolution workflows and computational runtime

To assess computational feasibility and runtime on large, high-resolution spatial transcriptomics datasets, we analyzed Visium HD and Xenium data from human colorectal cancer tissue sections generated by de Oliveira et al. [51]. We applied the smoothing, clustering, and BD workflow to process 545,850 nonempty 8μm Visium HD bins and 339,681 nonempty Xenium cells from one P2CRC section per platform. The Visium HD workflow used 1,000 selected variable genes, while the Xenium workflow used all 422 imported genes. For both platforms, PCA and K=15 k-means clustering were fitted on separate 30,000-location subsets, followed by full-data projection and assignment (see Supplementary Methods). Relative BD for the smoothclust-derived labels was 0.181 for Visium HD and 0.186 for Xenium; for the 15 distributed Visium HD labels, it was 0.364 (Supplementary Figure S15). These examples demonstrate the feasibility of applying the workflow to full high-resolution sections using subset-based fitting for PCA and clustering. The BD values describe each label map under its platform-specific graph and sampling units.

Observed runtimes for the core workflows were 54.0 seconds for Visium HD and 16.2 seconds for Xenium (Supplementary Table S2; Supplementary Figure S16). For the 19-section GBM analysis at K=5, the corresponding runtime was 33.9 seconds, reconstructed from component timers. We also compared runtimes across clustering methods in the GBM dataset. Under the evaluated settings, smoothclust, BANKSY, ClustSIGNAL, and BayesSpace required 33.9 seconds, 31.1 seconds, 52.2 seconds, and 1.72 hours, respectively (Supplementary Figure S16). Further timing details are provided in Supplementary Table S2 and Supplementary Figure S16.

## 3 Discussion

The smoothclust workflow and package provide a framework for translating categorical spatial maps into local and sample-level summaries of tissue organization. The framework combines a spatial-smoothing-based clustering approach for generating domain labels with a BD metric that can be calculated independently using labels from other algorithms, published annotations, or manual annotations. A key strength of BD is that it provides a simple, interpretable sample-level summary measure for exploratory comparisons across tissue sections, while retaining a direct connection to local label transitions and offering an optional composition adjustment based on expected transitions under random relabeling with the observed label counts. Our contribution combines an accessible R/Bioconductor implementation with analyses across spatial transcriptomics technologies, illustrating how BD summaries depend on label composition, label definitions, and spatial scale.

The applications illustrate several distinct uses of the BD metric. The GBM/glioma analyses quantify the effect of spatial smoothing on label organization and demonstrate the use of BD with labels generated by smoothclust and external clustering algorithms, as well as published tissue annotations, including comparisons among regional sections from the same tumor. The myocardial example uses published cell-type-niche labels for exploratory comparisons across author-defined tissue categories, including sections with strongly imbalanced label compositions. The cortical examples show how merging related annotation labels can change both the magnitude and ranking of section-level BD summaries, while manual anatomical annotations provide a separate example of agreement in relative BD between adjacent sections. Together, these analyses illustrate how BD can support different descriptive questions across datasets and biological contexts.

Raw and relative BD provide complementary summaries of tissue organization and should be interpreted together. Raw BD describes the observed frequency of label transitions, whereas relative BD expresses this frequency relative to its expectation under random relabeling with the observed label counts. Both quantities are informative: differences in label composition may themselves be biologically meaningful, and the adjustment does not make relative BD independent of composition in spatially structured maps. Both measures also depend on label definitions, graph construction, and spatial scale, so comparisons across tissue sections require comparable labels, neighborhoods, and sampling units. BD therefore provides an interpretable summary of overall label transitions at the chosen spatial scale, complementing more detailed analyses of boundary geometry and interactions between specific labels.

Spatial smoothing introduces a separate choice of scale. The strength of spatial smoothing should be selected with the biological context and intended spatial resolution in mind, since excessive smoothing may obscure small but biologically meaningful structures, as illustrated by the over-smoothing simulation. The default uniform-radius smoother uses a bandwidth proportional to each section’s coordinate extent, so the same nominal bandwidth can correspond to different physical smoothing radii across sections. Where a common physical scale is important, section-specific bandwidths can be chosen accordingly; an absolute-distance bandwidth option could further simplify this choice. Tissue-constrained smoothing neighborhoods may also help avoid averaging expression across gaps within tissue sections.

An important future direction is to apply BD in larger spatial transcriptomics studies with biological replication, enabling investigations of associations between tissue organization and clinical or experimental outcomes. Such analyses could use statistical models that account for patient or donor identity, distinguishing variation between individuals from variation among sections sampled from the same individual. BD could also serve as an exploratory sample-level diagnostic to identify sections with unusual spatial organization that warrant further investigation. Further evaluation of sensitivity to annotation uncertainty, graph construction, and tissue sampling would help establish the reliability of BD summaries in these settings.

Overall, smoothclust provides a transparent and reusable framework for characterizing spatial domain organization and tissue architecture through interpretable local and sample-level summaries of label transitions. The Visium HD and Xenium analyses further demonstrate the feasibility of applying the workflow to tissue sections containing hundreds of thousands of spatial locations on a laptop. By linking local visualization with sample-level summaries and illustrating how label composition, label definitions, and spatial scale influence BD, the framework supports exploratory analyses and comparisons of tissue organization across sections.

## 4 Methods

### 4.1 Framework and spatial smoothing

The smoothclust framework consists of two components: a spatial-smoothing-based clustering workflow and the boundary density (BD) metric. The clustering workflow normalizes gene expression counts, smooths normalized expression profiles across spatial neighbors, and log-transforms them before downstream clustering, while BD summarizes label transitions on a spatial graph. BD provides local and sample-level summaries for each tissue section and can be calculated independently of the smoothing workflow, using labels from smoothclust, external clustering algorithms, published annotations, or manual annotations.

Let $X\in R_{\geq 0}^{G\times N}$ contain expression counts for G genes and N spatial locations, and let $S\in R^{N\times 2}$ contain the matching spatial coordinates. Location- and gene-level filtering are performed before calling smoothclust(). If $q_{j}>0$ is the library total used for normalization at location j, the size factor, rescaled to have mean one, and normalized expression are

$$s_{j}=\frac{q_{j}}{N^{-1}\sum_{\ell =1}^{N}q_{\ell }},\;X_{gj}^{\text{norm}}=\frac{X_{gj}}{s_{j}}.$$

Normalization precedes smoothing so that neighboring locations contribute according to spatial weights rather than their library totals.

Spatial smoothing uses a sparse column-normalized weight matrix $W\in R^{N\times N}$, with $w_{ij}\geq 0$ and $\sum_{i}w_{ij}=1$, to calculate

$$X^{\text{smooth}}=X^{\text{norm}}W.$$

Here, $w_{ij}$ is the contribution of location i to the smoothed profile at location j. Averaging is performed on the linear normalized-expression scale. For the default uniform-radius smoother, let R be the larger horizontal or vertical range of the supplied coordinates and h=bR. Each profile is replaced by the equally weighted average of itself and all other locations within Euclidean distance h; the default bandwidth is b=0.05. The alternative kernel smoother uses exponentially decaying weights with length scale h, omitting unnormalized weights below τ=0.05. The k-nearest-neighbor smoother equally weights the focal location and $k_{s}$ neighbors, with default $k_{s}=18$. The smoothing-neighbor count $k_{s}$ is distinct from the BD neighborhood size k. Full weight definitions are provided in Supplementary Methods.

The default post-smoothing transformation is

$$Y={\text{log}}_{2}\left(X^{\text{norm}}W+1\right).$$

Feature selection, dimension reduction, and clustering follow the dataset-specific workflows described below, separating smoothing from the choice of clustering algorithm. Where the smoothing workflow was applied, normalization, smoothing, and clustering were performed separately within each tissue section. Analyses of published or manual annotations calculated BD directly from the supplied labels.

### 4.2 Boundary density metric

The BD metric is a graph-based summary measure of label transitions in a specified categorical label map. Let $c_{i}$ be the label at location i, and let ${𝒩}_{i}$ be its spatial neighborhood, excluding i, with $d_{i}=\left|{𝒩}_{i}\right|>0$. The local raw BD value is

$${\text{BD}}_{\text{raw,}i}=\frac{1}{d_{i}}\sum_{j\in {𝒩}_{i}}\text{I}\left(c_{i}\neq c_{j}\right),$$

and the sample-level raw BD value is

$${\text{BD}}_{\text{raw}}=\frac{1}{N}\sum_{i=1}^{N}{\text{BD}}_{\text{raw,}i}.$$

The boundary_density() function uses a directed Euclidean k-nearest-neighbor graph, with $d_{i}=k$ for every location and default k=6. Raw BD ranges from 0, when all neighboring labels agree, to 1, when every directed relation joins different labels.

Nearest neighbors were identified using BiocNeighbors::findKNN() with default search settings, independently of the domain labels. When equidistant candidates spanned the k-th-neighbor cutoff, the subset returned by the neighbor-search implementation was retained. Ties that only change the ordering of retained neighbors do not affect BD.

Raw BD measures label-transition frequency on a spatial graph rather than physical boundary length. Local BD highlights locations with frequent label transitions, while the sample-level mean weights each spot, bin, or cell equally. The default k=6 neighborhood approximates immediate neighbors in the interior of a complete Visium lattice, although nearest-neighbor graphs may span tissue gaps or sparsely sampled regions. Comparisons therefore require comparable label definitions, graph construction, spatial scale, and sampling units.

Because raw BD also depends on label composition, we provide an optional adjustment that produces relative BD. For L observed labels, let $n_{\ell }$ be the number of locations carrying label ℓ, out of N total locations. The expected raw BD under random relabeling with the observed label counts is

$${\text{BD}}_{0}=1-\sum_{\ell =1}^{L}\frac{n_{\ell }\left(n_{\ell }-1\right)}{N(N-1)}.$$

The spatial locations, graph, and label counts are held fixed while labels are reassigned uniformly among locations. Under this scheme, every ordered pair of distinct locations has the same probability of carrying different labels, yielding the expression above without requiring permutation simulations. For L equally abundant labels, the expectation is N(1-1/L)/(N-1), and therefore increases with the number of labels at fixed N. More generally, the expectation depends on the complete label composition rather than the number of labels alone. Splitting a label into nonempty categories increases the expectation, whereas merging labels decreases it whenever at least two of the merged labels are present.

Relative BD is

$$\text{rBD}=\frac{{\text{BD}}_{\text{raw}}}{{\text{BD}}_{0}},\;{\text{BD}}_{0}>0.$$

Values below, near, or above 1 indicate fewer, similar, or more label transitions than expected under random relabeling. Relative BD is undefined for single-label samples and may be unstable when expected raw BD is close to zero; a value near 1 does not establish spatial randomness.

Raw and relative BD provide complementary summaries: raw BD describes observed transition frequency, while relative BD expresses it relative to the random-label expectation. The adjustment accounts for label composition through this expectation but does not make relative BD independent of composition, label definitions, or spatial scale. Raw BD, expected raw BD, and relative BD should therefore be considered together.

### 4.3 Computational implementation and runtime

Analyses used R version 4.6.1 and smoothclust version 1.9.6. The smoothclust() function accepts a SpatialExperiment object [52] or an expression matrix with matching coordinates, uses sparse matrix operations and BiocNeighbors parallelization, and returns processed expression values with unchanged dimensions and names. For SpatialExperiment inputs, the processed values are added as a new assay and preprocessing settings are recorded in object metadata.

The boundary_density() function operates independently on spatial coordinates and categorical labels. Labels must be nonmissing, and the neighbor count must satisfy 1≤k<N. Its default output contains local and sample-level raw BD values; adjust = TRUE additionally returns expected raw BD and relative BD values at the sample level. Input handling and normalization are summarized in Supplementary Methods; detailed input requirements are provided in the R package documentation.

Runtime was measured on a 2023 MacBook Pro with an Apple M3 Max processor and 36 GB memory. Timing scope, requested threads, and the distinction between component-derived and directly observed timings are described in Supplementary Table S2 and Supplementary Figure S16.

### 4.4 Datasets and empirical analysis design

We analyzed publicly available Visium, Visium HD, and Xenium datasets using labels generated by clustering methods or obtained from published computational or manual annotations (Supplementary Table S1). BD was calculated separately for each tissue section. Repeated sections or regions from the same donor were identified in descriptive comparisons and were not treated as independent biological replicates. We used BD to characterize tissue organization in datasets from several platforms, interpreting values within each analysis in the context of differences in graph scale, spatial resolution, sampling units, and label granularity. Detailed preprocessing, feature selection, annotation matching, and coordinate transformations are provided in Supplementary Methods.

#### 4.4.1 Greenwald GBM/glioma Visium analyses

Greenwald et al. GBM/glioma Visium data were imported from GEO accession GSE237183 [47]. The clustering cohort comprised 13 GBM and six IDH-mutant glioma sections. After spot- and gene-level filtering, 34,699 of 39,566 spots and a shared set of 14,301 genes were retained across 19 sections.

Each section was analyzed independently. The primary workflow used uniform-radius smoothing with b=0.05, followed by variable-gene selection, PCA with 50 components, and k-means clustering with 25 random starts for K=3,…,8. A matched baseline omitted smoothing before log transformation. Both workflows used the same normalization and downstream analysis procedures, with variable genes and principal components estimated separately from the smoothed and unsmoothed expression matrices.

Local and sample-level BD were calculated using k=6. Sensitivity analyses compared uniform, kernel, and nearest-neighbor smoothing; varied smoothing parameters; and evaluated BD neighborhood sizes from k=2 to 36 while holding labels fixed. Agreement between clusterings obtained using different smoothing methods was assessed using the adjusted Rand index (ARI).

For the external-method comparison, BayesSpace and BANKSY were run with five clusters per section, while ClustSIGNAL used its default labeling settings and returned a variable number of clusters [9, 17, 18]. All methods used the same retained spots and shared gene set. Raw BD was calculated for each resulting label map using k=6.

We also analyzed four sets of published Greenwald annotation labels available for 13 GBM sections from five tumors: coarse organization zones (org1), malignancy-refined organization zones (org2), dominant metaprograms (mp), and deposited layer annotations (layer). Author-provided labels were retained as deposited. BD was calculated using k=6 on spots with nonmissing labels for each field, with relative BD used for sample-level comparisons across annotation types.

Finally, we compared metaprogram composition and relative BD for metaprogram and layer annotations descriptively across 12 regional sections from four tumors, including five sections from tumor ZH881.

#### 4.4.2 Kuppe myocardial-infarction Visium analyses

Processed Visium objects were obtained from the Human Cell Atlas (project e9f36305-d857-44a3-93f0-df4e6007dc97), with clinical metadata from Kuppe et al. [48]. The dataset contained 91,517 spots across 28 sections from 20 patients. We used the nine published computational cell-type-niche labels and retained the author-defined tissue categories: 14 myogenic-enriched, nine ischaemic-enriched, and five fibrotic-enriched sections. These categories describe tissue histomorphology; the myogenic-enriched category includes control tissue and remote or border regions from infarcted hearts. Array positions were transformed to an equilateral hexagonal coordinate system, and local and sample-level BD were calculated separately for each section using k=6.

Relative BD was the primary sample-level summary. Sections in which one niche occupied more than 80% of spots were flagged, and group medians were also calculated after excluding these sections. Comparisons were descriptive, with each section contributing equally to group summaries and repeated patient identities indicated in the plots.

Neighborhood-size sensitivity was assessed using the same published niche labels and all 28 retained sections, as described in Supplementary Methods and Supplementary Figure S11.

#### 4.4.3 Miyoshi frontal-cortex Visium analyses

The author-processed Miyoshi frontal-cortex object was obtained from GEO accession GSE233208 and contained 115,451 spots across 39 sections [49]. The cohort comprised 10 cognitively normal controls, nine early-stage Alzheimer’s disease (AD), 10 late-stage AD, and 10 Down-syndrome-associated AD samples. We analyzed the published BayesSpace q=9 annotation, comprising six cortical and three white-matter regions, and a second label definition obtained by merging WM1–WM3 into a single white-matter label while retaining the six cortical labels. Both definitions used the same retained spots, spot order, transformed Visium coordinates, and directed k=6 nearest-neighbor search settings within each section, excluding the focal spot. We compared local raw BD maps and sample-level raw BD, expected raw BD, and relative BD values, with the analytic expectation calculated from the label counts for each definition. Sensitivity to label granularity was assessed using paired changes in raw BD, expected raw BD, and relative BD and the Spearman correlation of section-level relative BD between the two definitions. The representative section was selected as the 20th of 39 sections ordered by increasing relative BD under the original annotation.

Neighborhood-size sensitivity was assessed using the original nine-region q=9 annotation and all 39 retained sections, as described in Supplementary Methods and Supplementary Figure S11.

#### 4.4.4 Maynard DLPFC Visium analyses

The Maynard dorsolateral prefrontal cortex (DLPFC) data were obtained from the spatialLIBD Bioconductor package [50, 53]. We analyzed 12 sections from three donors, retaining 47,329 spots with published manual cortical-layer (L1–L6) and white-matter annotations after excluding 352 unlabeled spots. Local and sample-level BD were calculated separately for each section using k=6. Six directly adjacent section pairs were identified using the deposited donor, within-block position, and replicate fields, and absolute differences in relative BD within pairs were summarized descriptively.

#### 4.4.5 de Oliveira colorectal-cancer Visium HD and Xenium analyses

P2CRC Visium HD and Xenium data from de Oliveira et al. [51] were obtained through the OSTA.data resource [5]. The analyses included 545,850 nonempty 8μm Visium HD bins and 339,681 nonempty Xenium cells from one section per platform. For Visium HD, 1,000 variable genes were selected before smoothing; Xenium analyses used all 422 imported genes. Expression was normalized and smoothed over $k_{s}=18$ neighbors across all retained locations, followed by log transformation. For each platform, PCA with 15 components and k-means clustering with K=15 were fitted on a randomly selected 30,000-location subset; all locations were then projected into the fitted PC space and assigned to the nearest cluster centroid. Local and sample-level BD were calculated using k=8 for Visium HD and k=6 for Xenium. BD was also calculated for the 15 Visium HD labels distributed with the source object. Feature-selection and fitting subsets, normalization, and full-data projection are detailed in Supplementary Methods.

### 4.5 Simulation designs

The composition simulation used 900 locations on a 30 × 30 grid and a directed four-nearest-neighbor graph. We generated 75 random arrangements at each exact 10:90, 30:70, and 50:50 two-label composition. Five additional maps with 450 locations per label compared coherent domains, partial label exchange, random placement, and a checkerboard. Three further maps contained contiguous domains at the same three compositions while retaining the same graph, interface orientation, and interface extent. Raw, expected, and relative BD were calculated for every map, with expectations obtained analytically from the label counts.

The over-smoothing simulation used 3,600 locations on a 60 × 60 grid with two broad domains and an 81-location circular island. We compared no smoothing with uniform bandwidths from 2.5% to 30% of the coordinate range, holding the selected feature set, number of principal components (10), and number of clusters (K=3) fixed. PCA was recomputed for each expression matrix. Domain recovery was assessed using ARI and island F1, and raw and relative BD were calculated using a directed four-nearest-neighbor graph. This analysis used one illustrative count realization. Count-generation procedures, label-exchange rules, complete simulation settings, and recovery-score definitions are provided in Supplementary Methods.

## Supplementary Material

1

## Figures and Tables

**Figure 1:**
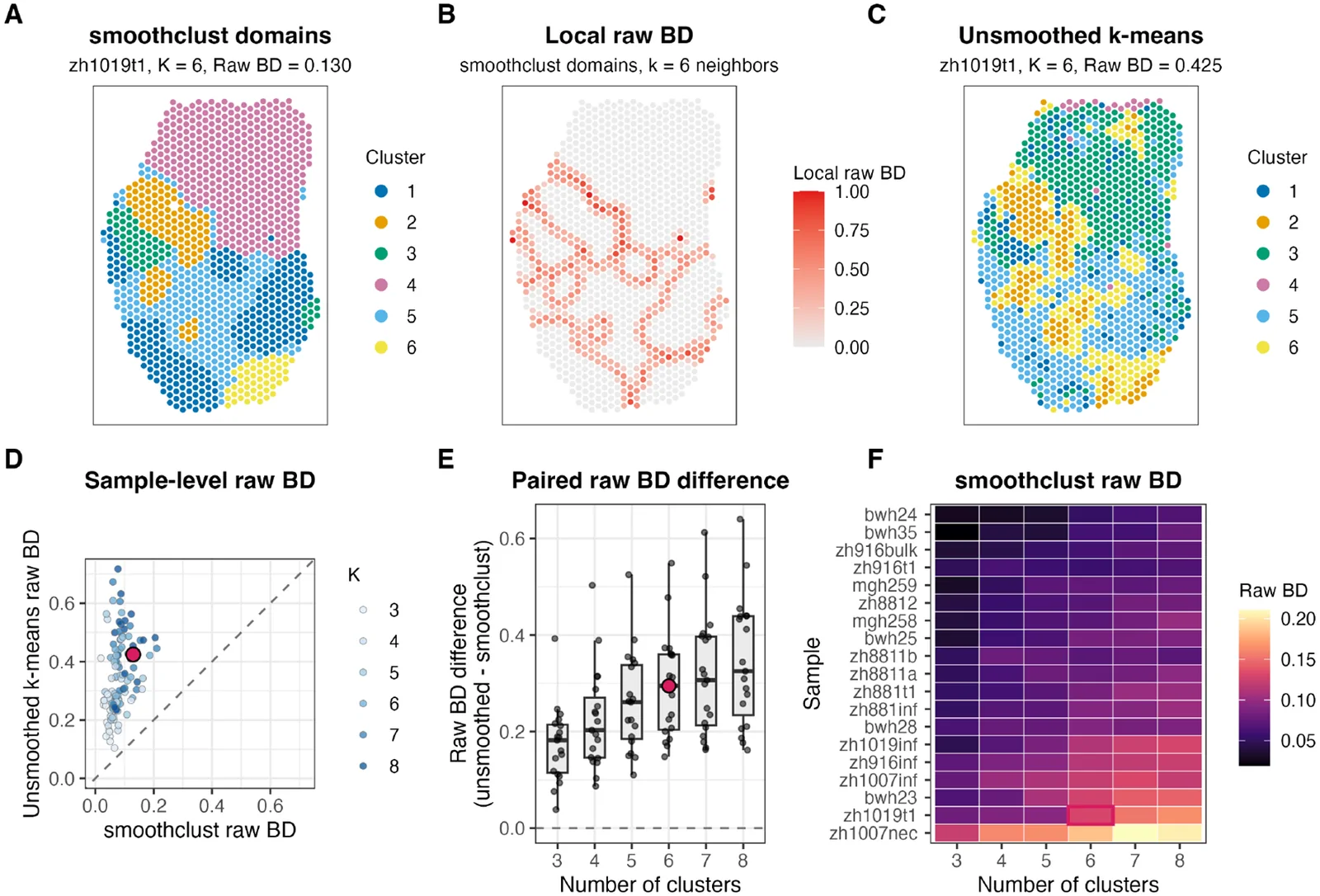
The BD metric summarizes the effect of spatial smoothing on GBM/glioma label maps. (A) smoothclust domain labels for section zh1019t1 with K=6. (B) Corresponding local raw BD map for the smoothclust labels. (C) Domain labels generated using the matched non-spatial k-means baseline without smoothing. (D) Paired sample-level raw BD values across 19 sections and K=3,…,8; the dashed line indicates equality. (E) Paired differences in raw BD (baseline minus smoothclust), grouped by cluster number. (F) Sample-level raw BD for smoothclust across sections and cluster numbers. The highlighted points and tile in panels D–F mark zh1019t1 at K=6. All BD calculations used k=6. Feature selection and PCA were performed separately for the smoothed and unsmoothed workflows.

**Figure 2:**
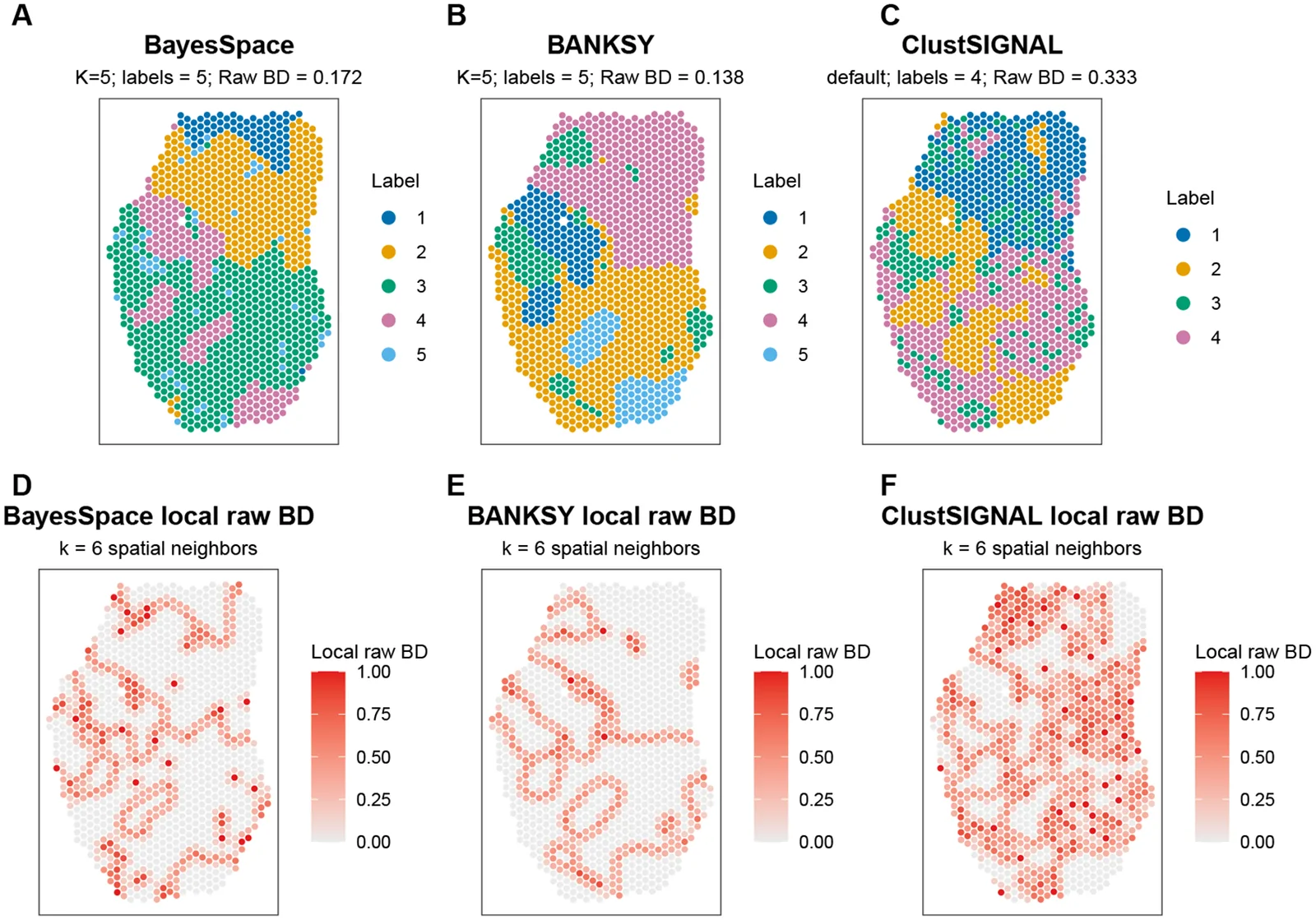
The BD metric summarizes labels generated using external spatial clustering algorithms. (A–C) Domain labels obtained by applying BayesSpace [9], BANKSY [17], and ClustSIGNAL [18], respectively, to section zh1019t1. (D–F) Corresponding local raw BD maps on a common 0–1 scale. Colors and cluster numbers do not imply matched biological identities across methods. BayesSpace and BANKSY used K=5; ClustSIGNAL used its default settings and returned four labels in this section. All BD calculations used k=6.

**Figure 3:**
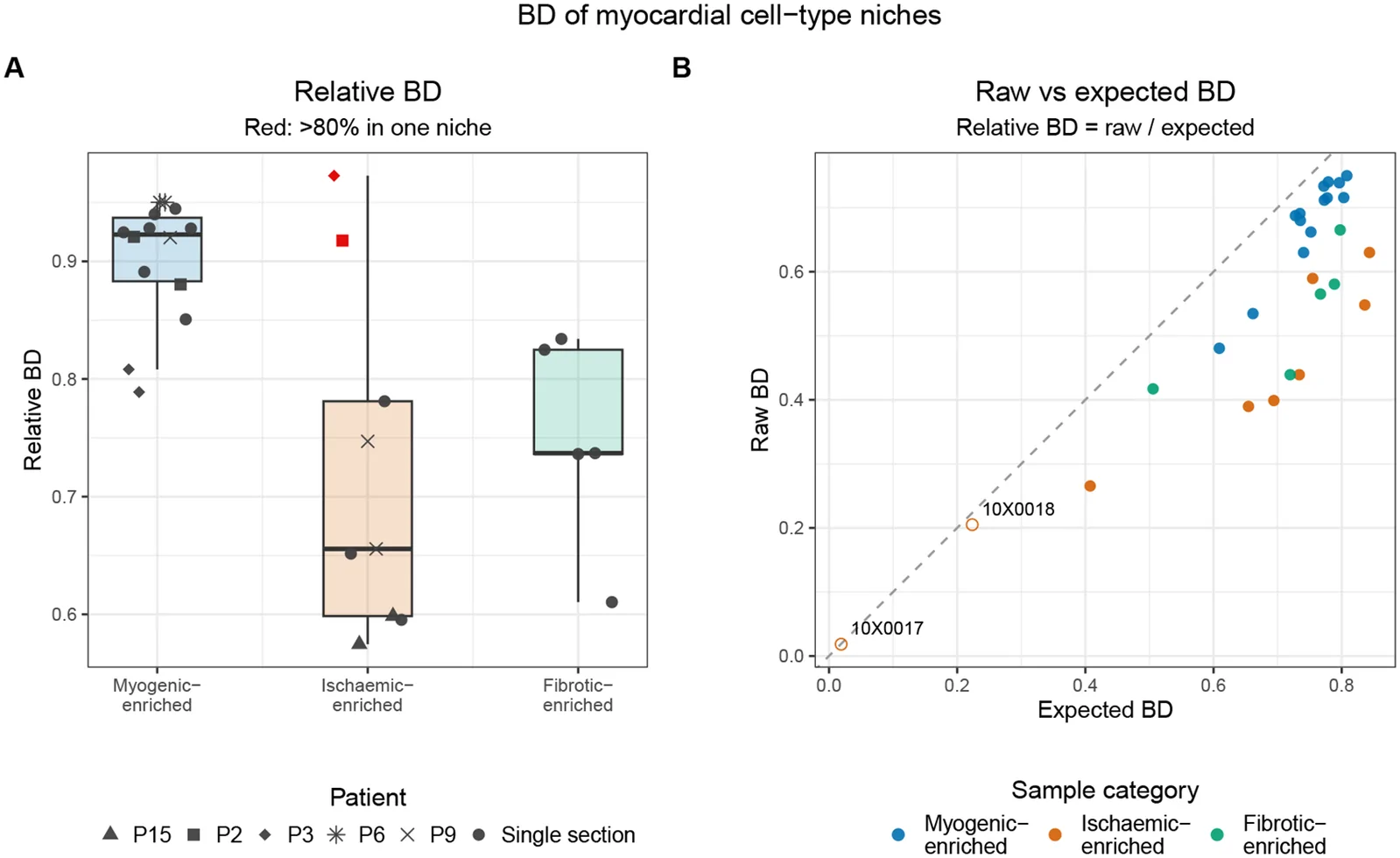
Relative BD summarizes published myocardial cell-type-niche organization. (A) Relative BD for the nine published cell-type-niche labels from Kuppe et al. [48] across 28 sections from 20 patients, grouped by the author-defined tissue categories: myogenic-enriched (14 sections), ischaemic-enriched (nine), and fibrotic-enriched (five). Each point is one section; boxes show medians and interquartile ranges and are colored by sample category. Shapes identify patients with multiple sections; circles denote patients with a single section. Red points mark two ischaemic sections in which one niche occupied more than 80% of spots. (B) Observed sample-level raw BD plotted against expected raw BD under random relabeling with the observed label counts in each section; the dashed line denotes equality. Point colors match the box colors in (A), and open circles mark the same two flagged sections. All BD calculations used k=6. Group summaries are descriptive and section-weighted.

**Figure 4:**
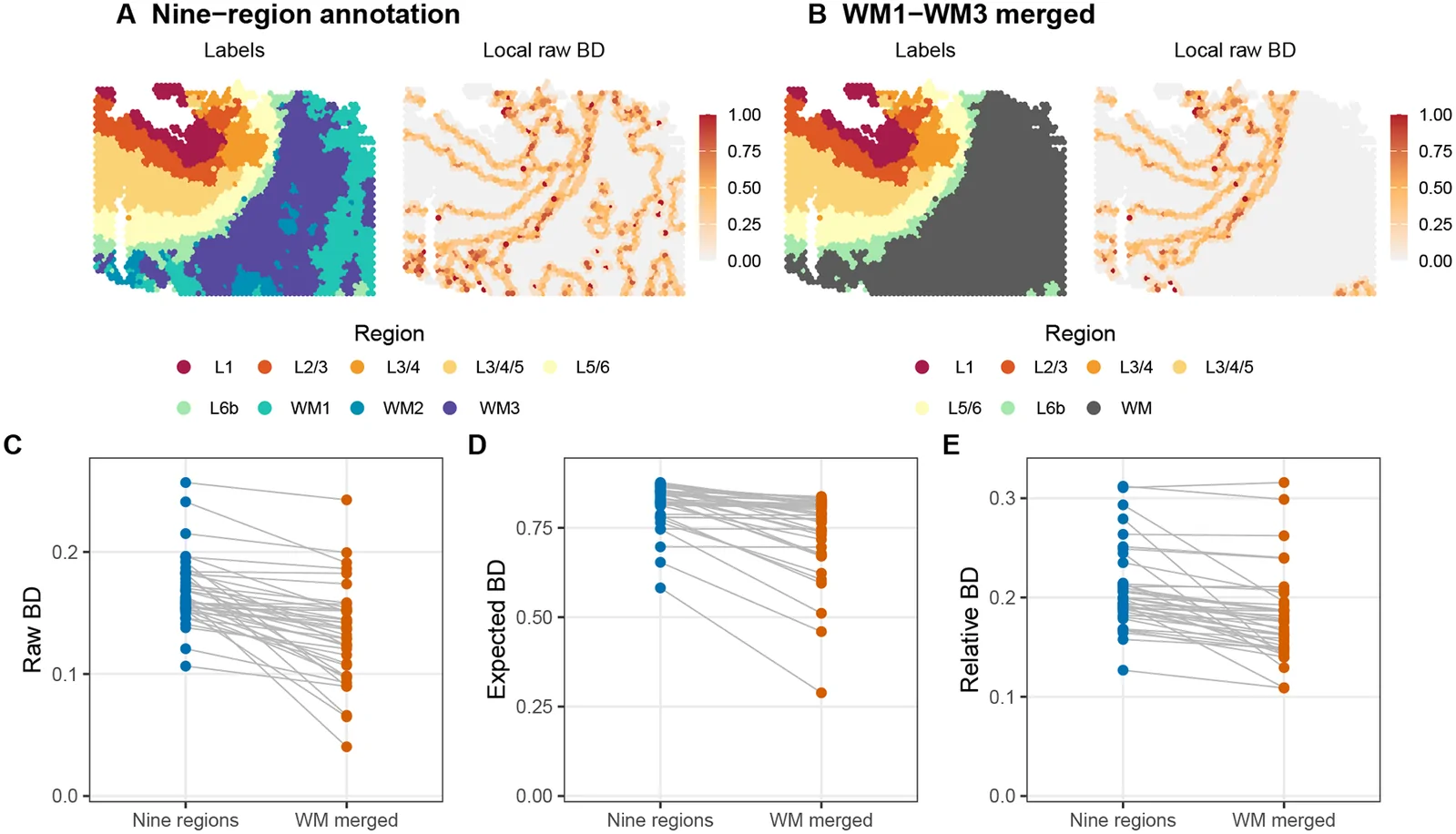
Raw, expected, and relative BD summarize the effects of cortical annotation granularity. (A) Published nine-region BayesSpace annotation and corresponding local raw BD map for a representative Miyoshi frontal-cortex Visium section (Nov_24_2021_VisiumHuman_12), selected as the 20th of 39 sections ordered by increasing relative BD under the original annotation. (B) The same section after merging WM1–WM3 into a single white-matter label, with the corresponding local raw BD map. (C) Paired sample-level raw BD values across all 39 sections under the original and merged annotation definitions. (D) Corresponding expected raw BD under random relabeling with the observed label counts, calculated analytically for each section and label definition. (E) Corresponding relative BD, calculated as raw BD divided by expected raw BD. Lines in C–E connect values from the same section. Retained spots, spot order, transformed coordinates, and directed k=6 neighbor-search settings were the same for both definitions; only the label definition changed. Local raw BD maps in A–B use a common 0–1 color scale.

## Data Availability

The smoothclust R package is available from Bioconductor at https://bioconductor.org/packages/smoothclust, and its source code is maintained at https://github.com/lmweber/smoothclust. Scripts to reproduce the preprocessing, analyses, figures, and runtime evaluations are available at https://github.com/lmweber/smoothclust-analyses. All datasets analyzed are publicly available from the repositories and accessions specified in the Methods and Supplementary Table S1.

## References

[1] Moses L and Pachter L. Museum of spatial transcriptomics. Nature Methods 2022; 19:534–46. doi: 10.1038/s41592-022-01409-2 35273392

[2] Tian L, Chen F, and Macosko EZ. The expanding vistas of spatial transcriptomics. Nature Biotechnology 2023; 41:773–82. doi: 10.1038/s41587-022-01448-2 PMC10091579 36192637

[3] Williams CG, Lee HJ, Asatsuma T, Vento-Tormo R, and Haque A. An introduction to spatial transcriptomics for biomedical research. Genome Medicine 2022; 14:68. doi: 10.1186/s13073-022-01075-1 PMC9238181 35761361

[4] Park J, Kim J, Lewy T, Rice CM, Elemento O, Rendeiro AF, and Mason CE. Spatial omics technologies at multimodal and single cell/subcellular level. Genome Biology 2022; 23:256. doi: 10.1186/s13059-022-02824-6 PMC9746133 36514162

[5] Crowell HL, Dong Y, Billato I, Cai P, Emons M, Gunz S, Guo B, Li M, Mahmoud A, Manukyan A, Pagès H, Panwar P, Rao S, Sargeant CJ, Shepherd Kern L, Ramos M, Sun J, Totty M, Carey VJ, Chen Y, Collado-Torres L, Ghazanfar S, Hansen KD, Martinowich K, Maynard KR, Patrick E, Righelli D, Risso D, Tiberi S, Waldron L, Gottardo R, Robinson MD, Hicks SC, and Weber LM. Orchestrating Spatial Transcriptomics Analysis with Bioconductor. bioRxiv 2026. doi: 10.1101/2025.11.20.688607

[6] Palla G, Spitzer H, Klein M, Fischer D, Schaar AC, Kuemmerle LB, Rybakov S, Ibarra IL, Holmberg O, Virshup I, Lotfollahi M, Richter S, and Theis FJ. Squidpy: a scalable framework for spatial omics analysis. Nature Methods 2022; 19:171–8. doi: 10.1038/s41592-021-01358-2 PMC8828470 35102346

[7] Hao Y, Stuart T, Kowalski MH, Choudhary S, Hoffman P, Hartman A, Srivastava A, Molla G, Madad S, Fernandez-Granda C, and Satija R. Dictionary learning for integrative, multimodal and scalable single-cell analysis. Nature Biotechnology 2024; 42:293–304. doi: 10.1038/s41587-023-01767-y PMC10928517 37231261

[8] Chen JG, Chávez-Fuentes JC, O’Brien M, Xu J, Ruiz EC, Wang W, Amin I, Sheridan JP, Shin SC, Hasyagar SV, Sarfraz I, Guckhool P, Sistig A, Jarzabek V, Yuan GC, and Dries R. Giotto Suite: a multiscale and technology-agnostic spatial multiomics analysis ecosystem. Nature Methods 2025; 22:2052–64. doi: 10.1038/s41592-025-02817-w PMC12510873 41034612

[9] Zhao E, Stone MR, Ren X, Guenthoer J, Smythe KS, Pulliam T, Williams SR, Uytingco CR, Taylor SEB, Nghiem P, Bielas JH, and Gottardo R. Spatial transcriptomics at subspot resolution with BayesSpace. Nature Biotechnology 2021; 39:1375–84. doi: 10.1038/s41587-021-00935-2 PMC8763026 34083791

[10] Li Z and Zhou X. BASS: multi-scale and multi-sample analysis enables accurate cell type clustering and spatial domain detection in spatial transcriptomic studies. Genome Biology 2022; 23:168. doi: 10.1186/s13059-022-02734-7 PMC9351148 35927760

[11] Shang L and Zhou X. Spatially aware dimension reduction for spatial transcriptomics. Nature Communications 2022; 13:7203. doi: 10.1038/s41467-022-34879-1 PMC9684472 36418351

[12] Liu W, Liao X, Luo Z, Yang Y, Lau MC, Jiao Y, Shi X, Zhai W, Ji H, Yeong J, and Liu J. Probabilistic embedding, clustering, and alignment for integrating spatial transcriptomics data with PRECAST. Nature Communications 2023; 14:296. doi: 10.1038/s41467-023-35947-w PMC9849443 36653349

[13] Hu J, Li X, Coleman K, Schroeder A, Ma N, Irwin DJ, Lee EB, Shinohara RT, and Li M. SpaGCN: Integrating gene expression, spatial location and histology to identify spatial domains and spatially variable genes by graph convolutional network. Nature Methods 2021; 18:1342–51. doi: 10.1038/s41592-021-01255-8 34711970

[14] Dong K and Zhang S. Deciphering spatial domains from spatially resolved transcriptomics with an adaptive graph attention auto-encoder. Nature Communications 2022; 13:1739. doi: 10.1038/s41467-022-29439-6 PMC8976049 35365632

[15] Long Y, Ang KS, Li M, Chong KLK, Sethi R, Zhong C, Xu H, Ong Z, Sachaphibulkij K, Chen A, Zeng L, Fu H, Wu M, Lim LHK, Liu L, and Chen J. Spatially informed clustering, integration, and deconvolution of spatial transcriptomics with GraphST. Nature Communications 2023; 14:1155. doi: 10.1038/s41467-023-36796-3 PMC9977836 36859400

[16] Pham D, Tan X, Balderson B, Xu J, Grice LF, Yoon S, Willis EF, Tran M, Lam PY, Raghubar A, Kalita-de Croft P, Lakhani S, Vukovic J, Ruitenberg MJ, and Nguyen QH. Robust mapping of spatiotemporal trajectories and cell-cell interactions in healthy and diseased tissues. Nature Communications 2023; 14:7739. doi: 10.1038/s41467-023-43120-6 PMC10676408 38007580

[17] Singhal V, Chou N, Lee J, Yue Y, Liu J, Chock WK, Lin L, Chang YC, Teo EML, Aow J, Lee HK, Chen KH, and Prabhakar S. BANKSY unifies cell typing and tissue domain segmentation for scalable spatial omics data analysis. Nature Genetics 2024; 56:431–41. doi: 10.1038/s41588-024-01664-3 PMC10937399 38413725

[18] Panwar P, Guo B, Zhou H, Hicks SC, and Ghazanfar S. ClustSIGNAL identifies cell types and subtypes using an adaptive smoothing approach for scalable spatial omics clustering. bioRxiv 2026. doi: 10.64898/2025.11.30.691081

[19] Yuan Z, Zhao F, Lin S, Zhao Y, Yao J, Cui Y, Zhang XY, and Zhao Y. Benchmarking spatial clustering methods with spatially resolved transcriptomics data. Nature Methods 2024; 21:712–22. doi: 10.1038/s41592-024-02215-8 38491270

[20] Hu Y, Xie M, Li Y, Rao M, Shen W, Luo C, Qin H, Baek J, and Zhou XM. Benchmarking clustering, alignment, and integration methods for spatial transcriptomics. Genome Biology 2024; 25:212. doi: 10.1186/s13059-024-03361-0 PMC11312151 39123269

[21] Korsunsky I, Millard N, Fan J, Slowikowski K, Zhang F, Wei K, Baglaenko Y, Brenner M, Loh PR, and Raychaudhuri S. Fast, sensitive and accurate integration of single-cell data with Harmony. Nature Methods 2019; 16:1289–96. doi: 10.1038/s41592-019-0619-0 PMC6884693 31740819

[22] Zeira R, Land M, Strzalkowski A, and Raphael BJ. Alignment and integration of spatial transcriptomics data. Nature Methods 2022; 19:567–75. doi: 10.1038/s41592-022-01459-6 PMC9334025 35577957

[23] Zhou X, Dong K, and Zhang S. Integrating spatial transcriptomics data across different conditions, technologies and developmental stages. Nature Computational Science 2023; 3:894–906. doi: 10.1038/s43588-023-00528-w 38177758

[24] Cao Y, Lin Y, Patrick E, Yang P, and Yang JYH. scFeatures: multi-view representations of single-cell and spatial data for disease outcome prediction. Bioinformatics 2022; 38:4745–53. doi: 10.1093/bioinformatics/btac590 PMC9563679 36040148

[25] Feng Y, Yang T, Zhu J, Li M, Doyle M, Ozcoban V, Bass GT, Pizzolla A, Cain L, Weng S, Pasam A, Kocovski N, Huang YK, Keam SP, Speed TP, Neeson PJ, Pearson RB, Sandhu S, Goode DL, and Trigos AS. Spatial analysis with SPIAT and spaSim to characterize and simulate tissue microenvironments. Nature Communications 2023; 14:2697. doi: 10.1038/s41467-023-37822-0 PMC10185501 37188662

[26] Canete NP, Iyengar SS, Ormerod JT, Baharlou H, Harman AN, and Patrick E. spicyR: spatial analysis of in situ cytometry data in R. Bioinformatics 2022; 38:3099–105. doi: 10.1093/bioinformatics/btac268 PMC9326848 35438129

[27] Ding DY, Tang Z, Zhu B, Ren H, Shalek AK, Tibshirani R, and Nolan GP. Quantitative characterization of tissue states using multiomics and ecological spatial analysis. Nature Genetics 2025; 57:910–21. doi: 10.1038/s41588-025-02119-z PMC11985343 40169791

[28] Tanevski J, Vulliard L, Ibarra-Arellano MA, Schapiro D, Hartmann FJ, and Saez-Rodriguez J. Learning tissue representation by identification of persistent local patterns in spatial omics data. Nature Communications 2025; 16:4071. doi: 10.1038/s41467-025-59448-0 PMC12044154 40307222

[29] Levy-Jurgenson A, Tekpli X, and Yakhini Z. Assessing heterogeneity in spatial data using the HTA index with applications to spatial transcriptomics and imaging. Bioinformatics 2021; 37:3796–804. doi: 10.1093/bioinformatics/btab569 PMC8598444 34358288

[30] Varrone M, Tavernari D, Santamaria-Martínez A, Walsh LA, and Ciriello G. CellCharter reveals spatial cell niches associated with tissue remodeling and cell plasticity. Nature Genetics 2024; 56:74–84. doi: 10.1038/s41588-023-01588-4 38066188

[31] Luo S, Germain PL, Meyenn F von, and Robinson MD. On metrics for subpopulation detection in single-cell and spatial omics data. Nucleic Acids Research 2025; 53:gkaf921. doi: 10.1093/nar/gkaf921 PMC12476229 41014637

[32] Reynoso S, Schiebout C, Krishna R, and Zhang F. STEAM: Spatial Transcriptomics Evaluation Algorithm and Metric for clustering performance. Briefings in Bioinformatics 2025; 26:bbaf570. doi: 10.1093/bib/bbaf570 PMC12576956 41171708

[33] Hu J, Du Y, Hou S, Ding Y, Li J, Wu H, and Sun X. CEMUSA: a graph-based integrative metric for evaluating clusters in spatial transcriptomics. Bioinformatics 2026; 42:btag056. doi: 10.1093/bioinformatics/btag056 PMC12960911 41663910

[34] Nnadi GO, Bonnici V, Avesani S, Viesi E, and Giugno R. A multiperspective evaluation framework of spatial transcriptomics clustering methods. NAR Genomics and Bioinformatics 2026; 8:lqag075. doi: 10.1093/nargab/lqag075 PMC13342736 42421749

[35] Moran PAP. The Interpretation of Statistical Maps. Journal of the Royal Statistical Society: Series B (Methodological) 1948; 10:243–51. doi: 10.1111/j.2517-6161.1948.tb00012.x

[36] Cliff AD and Ord JK. Spatial Processes: Models and Applications. London: Pion, 1981

[37] Newman MEJ. Mixing patterns in networks. Physical Review E 2003; 67:026126. doi: 10.1103/PhysRevE.67.026126 12636767

[38] Moran PAP. Notes on continuous stochastic phenomena. Biometrika 1950; 37:17–23. doi: 10.1093/biomet/37.1-2.17 15420245

[39] Geary RC. The contiguity ratio and statistical mapping. The Incorporated Statistician 1954; 5:115–41. doi: 10.2307/2986645

[40] Anselin L. Local indicators of spatial association - LISA. Geographical Analysis 1995; 27:93–115. doi: 10.1111/j.1538-4632.1995.tb00338.x

[41] Song J, Bassiouni R, and Craig D. stJoincount: Join count statistic for quantifying spatial correlation between clusters. R package 2026. doi: 10.18129/B9.bioc.stJoincount

[42] Moses L, Einarsson PH, Jackson K, Luebbert L, Booeshaghi AS, Antonsson S, Bray N, Melsted P, and Pachter L. Voyager: exploratory single-cell genomics data analysis with geospatial statistics. bioRxiv 2023. doi: 10.1101/2023.07.20.549945

[43] Sarkar S, Möller A, Hartebrodt A, Erdmann M, Ostalecki C, Baur A, and Blumenthal DB. Spatial cell graph analysis reveals skin tissue organization characteristic for cutaneous T cell lymphoma. npj Systems Biology and Applications 2024; 10:143. doi: 10.1038/s41540-024-00474-x PMC11612425 39622929

[44] Meyer-Bender M, Vöhringer H, Schniederjohann C, Koziel S, Chung E, Popova E, Brobeil A, Griese N, Kolks N, Held LM, Munir A, Scverse Community, Dietrich S, Bruch PM, and Huber W. Spatialproteomics: an interoperable toolbox for analyzing highly multiplexed fluorescence image data. Nature Methods 2026; 23:1564–73. doi: 10.1038/s41592-026-03155-1 PMC13441886 42498882

[45] Wang Y, Chen Y, Yang L, Wang C, Cai J, and Xin H. Benchmarking niche identification via domain segmentation for spatial transcriptomics data. bioRxiv 2026. doi: 10.64898/2026.02.27.708202

[46] He Y, Chen S, Ding L, He R, Peng X, Duan G, Zhu H, Li HD, Wang S, and Wang J. stEDGE enables edge-guided multiscale reconstruction of hierarchical spatial domains and transition interfaces in spatial transcriptomics. bioRxiv 2026. doi: 10.64898/2026.09.15.751915

[47] Greenwald AC, Darnell NG, Hoefflin R, Simkin D, Mount CW, Gonzalez Castro LN, Harnik Y, Dumont S, Hirsch D, Nomura M, Talpir T, Kedmi M, Goliand I, Medici G, Laffy J, Li B, Mangena V, Keren-Shaul H, Weller M, Addadi Y, Neidert MC, Suvà ML, and Tirosh I. Integrative spatial analysis reveals a multi-layered organization of glioblastoma. Cell 2024; 187:2485–2501.e26. doi: 10.1016/j.cell.2024.03.029 PMC11088502 38653236

[48] Kuppe C, Ramirez Flores RO, Li Z, Hayat S, Levinson RT, Liao X, Hannani MT, Tanevski J, Wünnemann F, Nagai JS, Halder M, Schumacher D, Menzel S, Schäfer G, Hoeft K, Cheng M, Ziegler S, Zhang X, Peisker F, Kaesler N, Saritas T, Xu Y, Kassner A, Gummert J, Morshuis M, Amrute J, Veltrop RJA, Boor P, Klingel K, Van Laake LW, Vink A, Hoogenboezem RM, Bindels EMJ, Schurgers L, Sattler S, Schapiro D, Schneider RK, Lavine K, Milting H, Costa IG, Saez-Rodriguez J, and Kramann R. Spatial multi-omic map of human myocardial infarction. Nature 2022; 608:766–77. doi: 10.1038/s41586-022-05060-x PMC9364862 35948637

[49] Miyoshi E, Morabito S, Henningfield CM, Das S, Rahimzadeh N, Kiani Shabestari S, Michael N, Emerson N, Reese F, Shi Z, Cao Z, Srinivasan SS, Scarfone VM, Arreola MA, Lu J, Wright S, Silva J, Leavy K, Lott IT, Doran E, Yong WH, Shahin S, Perez-Rosendahl M, Alzheimer’s Biomarkers Consortium–Down Syndrome (ABC-DS), Head E, Green KN, and Swarup V. Spatial and single-nucleus transcriptomic analysis of genetic and sporadic forms of Alzheimer’s disease. Nature Genetics 2024; 56:2704–17. doi: 10.1038/s41588-024-01961-x PMC11631771 39578645

[50] Maynard KR, Collado-Torres L, Weber LM, Uytingco C, Barry BK, Williams SR, Catallini II JL, Tran MN, Besich Z, Tippani M, Chew J, Yin Y, Kleinman JE, Hyde TM, Rao N, Hicks SC, Martinowich K, and Jaffe AE. Transcriptome-scale spatial gene expression in the human dorsolateral prefrontal cortex. Nature Neuroscience 2021; 24:425–36. doi: 10.1038/s41593-020-00787-0 PMC8095368 33558695

[51] de Oliveira MF, Romero JP, Chung M, Williams SR, Gottscho AD, Gupta A, Pilipauskas SE, Mohabbat S, Raman N, Sukovich DJ, Patterson DM, Visium HD Development Team, and Taylor SEB. High-definition spatial transcriptomic profiling of immune cell populations in colorectal cancer. Nature Genetics 2025; 57:1512–23. doi: 10.1038/s41588-025-02193-3 PMC12165841 40473992

[52] Righelli D, Weber LM, Crowell HL, Pardo B, Collado-Torres L, Ghazanfar S, Lun ATL, Hicks SC, and Risso D. SpatialExperiment: infrastructure for spatially-resolved transcriptomics data in R using Bioconductor. Bioinformatics 2022; 38:3128–31. doi: 10.1093/bioinformatics/btac299 PMC9154247 35482478

[53] Pardo B, Spangler A, Weber LM, Page SC, Hicks SC, Jaffe AE, Martinowich K, Maynard KR, and Collado-Torres L. spatialLIBD: an R/Bioconductor package to visualize spatially-resolved transcriptomics data. BMC Genomics 2022; 23:434. doi: 10.1186/s12864-022-08601-w PMC9188087 35689177

