# Supplementary material for: smoothclust: Spatial smoothing and boundary-density summaries for spatial transcriptomics
Source: bioRxiv. 2026 Oct 30:2026.10.26.761912. Preprint. [Version 1] doi: 10.64898/2026.10.26.761912 (PMC13733862; doi:10.64898/2026.10.26.761912)
Supplement: Supplementary file 1 [file NIHPP2026.10.26.761912V1-supplement-1.pdf]

bioRxiv preprint doi: https://doi.org/10.64898/2026.10.26.761912; this version posted October 30, 2026. The copyright holder for this preprint (which was not certified by peer review) is the author/funder, who has granted bioRxiv a license to display the preprint in perpetuity. It is made available under a CC-BY 4.0 International license.

Supplementary Materials

Supplementary Methods

Smoothing weights and spatial scale. Let $\boldsymbol{r}_i$ be row i of S, $d_{ij} = \|\boldsymbol{r}_i - \boldsymbol{r}_j\|_2$, and $h = bR$, where R is the larger horizontal or vertical coordinate range. Uniform-radius smoothing uses

$$w_{ij} = \frac{\mathrm{I}(d_{ij} \le h)}{\sum_{u=1}^{N} \mathrm{I}(d_{uj} \le h)}.$$

For exponential-kernel smoothing, with $h > 0$,

$$\widetilde{w}_{ij} = \exp(-d_{ij}/h)\,\mathrm{I}\{d_{ij} \le -h\log(\tau)\}, \qquad w_{ij} = \frac{\widetilde{w}_{ij}}{\sum_{u=1}^{N} \widetilde{w}_{uj}},$$

where unnormalized weights below $\tau = 0.05$ are omitted by default. For nearest-neighbor smoothing, let $\mathcal{M}_j$ contain the k_s nearest locations other than j. Then

$$w_{ij} = \frac{\mathrm{I}\{i \in \mathcal{M}_j \cup \{j\}\}}{k_s + 1}.$$

All three smoothers include the focal location. The default $k_s = 18$ corresponds to two neighbor layers in a complete Visium hexagonal layout. Equal nominal settings do not imply equal physical scales: bR is a hard radius for uniform smoothing but an exponential length scale for the kernel, whose retained support extends to approximately $3bR$ at $\tau = 0.05$. Nearest-neighbor smoothing fixes neighbor count; smoothing scale is distinct from the neighborhood used to calculate BD.

Input handling and normalization. Expression columns, coordinate rows, and labels were supplied in matching order, with tissue sections analyzed separately. Size factors were supplied explicitly or calculated from column sums of the selected expression matrix, then rescaled to mean one. Dataset-specific normalization references are given below.

Derivation of the random-label expectation. For a fixed graph without self-relations on $N > 1$ locations, let π uniformly reassign labels while preserving counts $n_1, \ldots, n_L$. Writing $p_\ell = n_\ell/N$, any pair $i \ne j$ satisfies

$$\Pr(c_i^\pi \ne c_j^\pi) = 1 - \sum_{\ell=1}^{L} \frac{n_\ell(n_\ell - 1)}{N(N-1)} = \frac{N}{N-1}\left(1 - \sum_{\ell=1}^{L} p_\ell^2\right) = \mathrm{BD}_0.$$

The factor $N/(N-1)$ accounts for sampling labels without replacement. Linearity of expectation, without requiring independent edge indicators, gives

$$\mathbb{E}_\pi[\mathrm{BD}_{\mathrm{raw},i}] = \mathbb{E}_\pi[\mathrm{BD}_{\mathrm{raw}}] = \mathrm{BD}_0.$$

bioRxiv preprint doi: https://doi.org/10.64898/2026.10.26.761912; this version posted October 30, 2026. The copyright holder for this preprint (which was not certified by peer review) is the author/funder, who has granted bioRxiv a license to display the preprint in perpetuity. It is made available under a CC-BY 4.0 International license.

Consequently, $\mathbb{E}_\pi[\mathrm{rBD}] = 1$ whenever $\mathrm{BD}_0 > 0$. The same expectation holds for fixed nonnegative weights on distinct location pairs normalized by their positive total. Observed BD and its random-label variability remain graph-dependent.

Merging labels with counts n_a and n_b, while retaining the same locations, changes the expectation by

$$\mathrm{BD}_{0,\mathrm{before}} - \mathrm{BD}_{0,\mathrm{after}} = \frac{2n_a n_b}{N(N-1)}.$$

The expectation decreases when both labels are present; repeated application gives the result for merging multiple labels. On an unchanged graph, neither raw BD nor its expectation can increase after merging, but relative BD can increase if its denominator decreases proportionally more than its numerator.

Relationship to SHouT graph homophily and focal-label agreement. For a directed k-nearest-neighbor graph, global edge homophily satisfies

$$H = \frac{1}{Nk}\sum_{i=1}^{N}\sum_{j\in\mathcal{N}_i} \mathrm{I}(c_i = c_j), \qquad \mathrm{BD}_{\mathrm{raw}} = 1 - H.$$

Equality with the complement of SHouT's global homophily requires matching neighbor relations, multiplicities, and weights, but not graph symmetry [43]. The fraction of neighbors sharing the focal label is $1-\mathrm{BD}_{\mathrm{raw},i}$ when the focal location is excluded. If egophily e_i includes the focal location and the same d_i neighbors, then $1 - e_i = \{d_i/(d_i+1)\}\mathrm{BD}_{\mathrm{raw},i}$. SHouT's local homophily instead summarizes edges within a neighborhood and is distinct from this focal-label comparison.

Relationship to Geary's C. For the directed k-nearest-neighbor graph, let $a_{ij} = \mathrm{I}(j \in \mathcal{N}_i)$, $a_{ii} = 0$, and $A_0 = \sum_{i,j} a_{ij} = Nk$. For nonconstant x, Geary's statistic is [39]

$$C(x) = \frac{N-1}{2A_0}\frac{\sum_{i,j} a_{ij}(x_i - x_j)^2}{\sum_i (x_i - \bar{x})^2}.$$

For at least two observed labels, define $z_{i\ell} = \mathrm{I}(c_i = \ell)$ and $v_\ell = \sum_i (z_{i\ell} - p_\ell)^2 = Np_\ell(1-p_\ell)$. Since $\sum_{\ell=1}^{L}(z_{i\ell} - z_{j\ell})^2 = 2\,\mathrm{I}(c_i \neq c_j)$,

$$\sum_{\ell:v_\ell>0} v_\ell C(z_\ell) = (N-1)\mathrm{BD}_{\mathrm{raw}}, \qquad \sum_{\ell=1}^{L} v_\ell = (N-1)\mathrm{BD}_0,$$

and therefore

$$\mathrm{rBD} = \frac{\sum_{\ell:v_\ell>0} v_\ell C(z_\ell)}{\sum_{\ell:v_\ell>0} v_\ell}.$$

Thus, relative BD is a variance-weighted, not generally unweighted, mean of indicator-specific Geary statistics. With two labels, the complementary indicators have equal Geary statistics, giving $\mathrm{rBD} = C(x)$ for either binary indicator x. These identities require matching locations and spatial weights. For a variable-degree graph with all $d_i > 0$, the corresponding mean of local fractions uses row-normalized weights $a_{ij} = \mathrm{I}(j \in \mathcal{N}_i)/d_i$, with $A_0 = N$.

bioRxiv preprint doi: https://doi.org/10.64898/2026.10.26.761912; this version posted October 30, 2026. The copyright holder for this preprint (which was not certified by peer review) is the author/funder, who has granted bioRxiv a license to display the preprint in perpetuity. It is made available under a CC-BY 4.0 International license.

Relationship to the initial stEDGE boundary signal. stEDGE constructs an initial boundary signal from consensus co-assignment across labelings [46]. For M labelings of the same locations, define

$$Q_{ij} = \frac{1}{M} \sum_{m=1}^{M} \mathrm{I}\left(c_i^{(m)} = c_j^{(m)}\right), \qquad i \neq j.$$

For a common neighborhood $\mathcal{N}_i$ excluding i, its complement-of-consensus score is

$$b_i = 1 - \frac{1}{d_i} \sum_{j \in \mathcal{N}_i} Q_{ij} = \frac{1}{M} \sum_{m=1}^{M} \mathrm{BD}_{\mathrm{raw},i}\left(c^{(m)}\right).$$

This identity requires matching neighborhoods, normalization, and exclusion of self-relations. It concerns only the initial boundary signal, not stEDGE's subsequent domain reconstruction, hierarchical organization, or transition index, and does not imply equivalence to relative BD.

GBM preprocessing and feature selection. GEO matrices were restricted to in-tissue spots with at least 1,000 total UMIs, at most 20% mitochondrial UMIs, finite totals and mitochondrial percentages, and positive nonmitochondrial totals. Genes with zero counts across retained spots were removed; the shared analysis set retained nonmitochondrial genes with at least 100 counts in at least one section, excluding spots with zero counts after filtering. Both workflows used size factors based on all nonmitochondrial genes before low-expression filtering, rescaled to mean one within sections, and pseudocount one. Features were selected separately from smoothed and unsmoothed log-expression using `scran::modelGeneVar()` and `scran::getTopHVGs()` with `prop=0.1` and otherwise default arguments; fitted scores ranked features for smoothed expression. PCA was fitted separately with `scater::runPCA()`, the selected genes, 50 components, and otherwise default settings.

External clustering settings. BayesSpace 1.22.0 used `spatialPreprocess()` with $\lceil 0.1G \rceil$ variable genes from the G shared input genes, 15 principal components, and log normalization with the above per-section size factors. Clustering used $q = 5$, k-means initialization, the t model with equal precision, 50,000 MCMC iterations, 1,000 burn-in iterations, and thinning interval 100 [9]. BANKSY (`Banksy` 1.8.1) and ClustSIGNAL 1.4.1 used `scuttle::logNormCounts()` with the same size factors and pseudocount one, followed by the above feature-selection procedure (`prop=0.1`). BANKSY used $M = 0$, $\lambda = 0.8$, $k_{\mathrm{geom}} = 15$, no azimuthal Gabor features, 50 principal components, and $K = 5$ k-means [17]. ClustSIGNAL used default labeling settings through `clustSIGNAL(spe_cs, samples="sample_id", threads=14, outputs="s")` [18]. Fourteen threads were requested where supported; effective parallelism could vary by stage. Seeds were deterministic by section.

Greenwald annotation matching and summaries. Annotations in `visium_metadata.csv` were matched by section identifier and spot barcode after mapping processed section names to deposited identifiers. Unmatched spots received missing annotations. Deposited labels, including layer annotations for Chromatin-Reg-dominant spots, were retained. Each annotation field used a graph of spots with nonmissing labels. Structured-zone fraction was the proportion of `org1`-labeled spots assigned `struct`;

bioRxiv preprint doi: https://doi.org/10.64898/2026.10.26.761912; this version posted October 30, 2026. The copyright holder for this preprint (which was not certified by peer review) is the author/funder, who has granted bioRxiv a license to display the preprint in perpetuity. It is made available under a CC-BY 4.0 International license.

MES-Hyp fraction was the proportion assigned `MES.Hyp` among spots with nonmissing `mp` within the `org1`-labeled subset. Metaprogram composition used all spots with nonmissing `mp` per section. Regional comparisons included 12 sections from ZH1007, ZH1019, ZH881, and ZH916. GBM graphs used `pxl_col_in_fullres` and `pxl_row_in_fullres` in full-resolution image pixels.

GBM smoothing and graph-scale sensitivity. Uniform and kernel smoothing at $b = 0.05$ were compared with nearest-neighbor smoothing at $k_s = 18$, without matching effective spatial reach. Parameter sensitivity used $b = 0.010, 0.025, 0.050, 0.075, 0.100, 0.150$ and $k_s = 2, 6, 12, 18, 30, 42$, respectively. BD neighborhood sensitivity held labels fixed and used $k = 2, 4, 6, 8, 12, 18, 24, 36$. All settings were evaluated independently by section for $K = 3, \ldots, 8$.

Kuppe and Miyoshi annotation matching and coordinates. Kuppe metadata, expression columns, and image coordinates required identical barcode order; deposited `celltype_niche` labels were retained. Miyoshi coordinates were matched to spot identifiers in metadata order, using labels from the deposited `annotation` field. For both datasets, array coordinates were transformed as $x = \texttt{col}/2$ and $y = \texttt{row}\sqrt{3}/2$. After excluding missing or empty labels, section-specific directed Euclidean graphs used $k = 6$, excluding focal locations, with `BiocNeighbors::findKNN()`, four requested threads, and otherwise default search settings.

Neighborhood-size sensitivity for published annotations. For the Kuppe nine-niche labels (28 sections) and Miyoshi nine-region BayesSpace annotation (39 sections), BD at $k = 18$ and $k = 36$ was compared with $k = 6$ (Supplementary Figure S11). Directed Euclidean graphs were rebuilt per section and neighborhood size, excluding focal locations while retaining the same spots, coordinates, labels, spot order, and search settings. Recomputed $k = 6$ values were checked against the saved baseline, and analytic expectations were verified unchanged. For raw and relative BD, summaries comprised Spearman correlations with $k = 6$, signed changes (larger k minus $k = 6$), and median and maximum absolute changes. Kuppe category medians and interquartile ranges used all sections with equal section weighting at each k; comparisons were descriptive, not treating repeated patient sections as independent replicates.

Maynard annotations. The processed `sce` object distributed through `spatialLIBD` (version 1.24.0) was used, as described in Supplementary Table S1. Labels were from `layer_guess_reordered_short`. All nonmissing, nonempty labels were retained, including 534 spots marked `discard=TRUE`; 352 unannotated spots were excluded. Adjacent pairs used `subject`, `position`, `replicate`, and `subject_position`, pairing replicates 1 and 2 within donor and within-block position.

High-resolution preprocessing and sketching. Visium HD and Xenium locations with positive imported count totals were retained; these full-library totals provided mean-one size factors. Visium HD genes were eligible if detected in at least 0.1% of retained bins, excluding mitochondrial genes. The 1,000 genes with highest variance of $\log_2(1 + \text{normalized counts})$ were selected on a random 25,000-bin subset, with library totals rescaled to mean one within that subset. Bins with zero counts among

bioRxiv preprint doi: https://doi.org/10.64898/2026.10.26.761912; this version posted October 30, 2026. The copyright holder for this preprint (which was not certified by peer review) is the author/funder, who has granted bioRxiv a license to display the preprint in perpetuity. It is made available under a CC-BY 4.0 International license.

selected genes were retained. Xenium used all 422 imported genes. Normalization, nearest-neighbor smoothing, and log transformation used all retained locations. PCA and clustering were fitted on a separate random 30,000-location subset for each platform; the Visium HD fitting subset was sampled separately from its feature-selection subset. All subsets were sampled without replacement. PCA used `irlba::prcomp_irlba()`, centering without scaling; all locations were projected using the fitted centering and loadings. k-means used three starts and at most 500 iterations, followed by nearest-centroid assignment of all locations. Visium HD coordinates were `array_col` and `array_row` (one unit $= 8\,\mu$m); Xenium used the imported spatial coordinates.

Composition simulation. The composition simulation used a 30×30 grid with exact A:B label counts of 90:810, 270:630, or 450:450. We generated 75 random label arrangements per composition. For the fixed 450:450 composition, a coherent map assigned A to $x \leq 15$ and B otherwise. To introduce partial mixing, we randomly exchanged labels at 10% or 30% of locations while preserving the label counts. We also generated a random arrangement and a checkerboard based on the parity of $x + y$. Additional straight-interface maps assigned A to $x \leq t$, with $t = 3, 9, 15$, and B otherwise, varying composition while retaining the same interface orientation and extent. All calculations used directed four-nearest-neighbor graphs without periodic boundaries.

Over-smoothing simulation. The over-smoothing simulation used 3,600 locations on a 60×60 grid with two broad domains and a small circular third-domain island containing 81 locations. For 120 genes, baseline means were drawn from a gamma distribution (shape and rate both 1.5), with 15 distinct marker genes per domain having twofold higher means. Negative-binomial counts (size parameter 1) were generated with location-specific log-normal library multipliers (log-standard deviation 0.35), rescaled to mean one. Counts were normalized using observed library totals. The 75 most variable genes from unsmoothed log-transformed normalized expression were selected and held fixed across settings. We compared an unsmoothed baseline with eight uniform-smoothing bandwidths ranging from 2.5% to 30% of the coordinate range. For each setting, PCA was recomputed, followed by $K = 3$ k-means clustering using the first 10 PCs. Domain recovery was assessed using the adjusted Rand index (ARI) and island F1, defined for true island I and inferred clusters C_j as

$$\max_j \frac{2|I \cap C_j|}{|I| + |C_j|}.$$

Counts, normalization factors, selected features, coordinates, and directed four-nearest-neighbor settings were held fixed across bandwidths. Cluster labels were aligned to the true labels only for visualization. This analysis used one illustrative count realization.

bioRxiv preprint doi: https://doi.org/10.64898/2026.10.26.761912; this version posted October 30, 2026. The copyright holder for this preprint (which was not certified by peer review) is the author/funder, who has granted bioRxiv a license to display the preprint in perpetuity. It is made available under a CC-BY 4.0 International license.

Supplementary Tables

Supplementary Table S1: Spatial transcriptomics datasets and analysis inputs used in this study.

Dataset	Reference and data source	Platform	Samples analyzed	Spatial locations	Expression inputs and labels
GBM/glioma	Greenwald et al. (2024) [47]; GEO GSE237183	Visium, fresh frozen	19 sections[a]	34,699 retained spots	14,301 shared input genes; smoothclust, baseline, and external-method labels; deposited `org1`, `org2`, `mp`, `layer`
Myocardial infarction	Kuppe et al. (2022) [48]; HCA[b]	Visium, fresh frozen	28 sections from 20 patients	91,517 annotated spots	Nine published cell-type-niche labels
Human frontal cortex	Miyoshi et al. (2024) [49]; GEO GSE233208[c]	Visium, fresh frozen	39 sections	115,451 deposited spots	Published nine-region BayesSpace annotation (six cortical, three white-matter regions) and WM1–WM3-merged definition
Human DLPFC	Maynard et al. (2021) [50]; `spatialLIBD` [53] archive[d]	Visium, fresh frozen	12 sections from 3 donors; 6 adjacent pairs	47,329 annotated spots	Published manual L1–L6 and white-matter labels
Visium HD CRC	de Oliveira et al. (2025) [51]; `OSTA.data`[e]	Visium HD, FFPE	One P2CRC section	545,850 nonempty 8 μm bins	1,000 selected variable genes; 15 smoothclust-derived domains; 15 distributed cluster labels
Xenium CRC	de Oliveira et al. (2025) [51]; `OSTA.data`[e]	Xenium In Situ, FFPE	One P2CRC section	339,681 nonempty segmented cells	All 422 imported genes; 15 smoothclust-derived domains

All platforms are from 10x Genomics. [a]The GBM/glioma clustering cohort comprised 13 GBM and six IDH-mutant glioma sections. Published annotations were available for 13 sections; regional analyses used 12 sections from four tumors. Annotation file: `Inputs/general/visium_metadata.csv`. [b]HCA project `e9f36305-d857-44a3-93f0-df4e6007dc97`; 28 sections represent 27 patient-region samples. [c]Processed object: `GSE233208_Human_visium_ADDS_seurat_processed.rds.gz`. [d]Archived processed object: `Human_DLPFC_Visium_processedData_sce_scran_spatialLIBD.Rdata`; annotation retention and software version are described in Supplementary Methods. [e]Serial sections from the same P2CRC FFPE block. `OSTA.data` resources: `VisiumHD_HumanColon_Oliveira` and `Xenium_HumanColon_Oliveira`. Visium HD inputs: `binned_outputs/square_008um/filtered_feature_bc_matrix.h5` and distributed labels in `clustering.csv.gz`; Xenium count input: `cell_feature_matrix.h5`.

bioRxiv preprint doi: https://doi.org/10.64898/2026.10.26.761912; this version posted October 30, 2026. The copyright holder for this preprint (which was not certified by peer review) is the author/funder, who has granted bioRxiv a license to display the preprint in perpetuity. It is made available under a CC-BY 4.0 International license.

Supplementary Table S2: Runtime for representative core smoothclust workflows.

Workflow	Sections	Locations	Input genes	Feature-reduction and clustering scope	Requested NN threads	Runtime
GBM Visium	19	34,699	14,301	Independent full-data workflow per section, $K = 5$	14	33.9 s
P2CRC Visium HD	1	545,850	1,000	30,000-bin PCA/clustering fit; full-data projection and assignment, $K = 15$	8	54.0 s
P2CRC Xenium	1	339,681	422	30,000-cell PCA/clustering fit; full-data projection and assignment, $K = 15$	8	16.2 s

Timings were measured on a 2023 MacBook Pro (Apple M3 Max, 14 cores, 36 GB memory). NN denotes nearest-neighbor searches; thread counts are requested values, and effective parallelism may vary. Core runtimes include smoothing, feature reduction, clustering or full-data assignment, and BD; they exclude data import, neighbor-distance diagnostics, file output, plotting, and the additional BD calculation for distributed Visium HD labels. GBM variable-gene selection and PCA were timed; Visium HD variable-gene selection occurred before timing, and Xenium used all 422 imported genes. The GBM $K = 5$ runtime was reconstructed from component timers in the $K = 3, \ldots, 8$ analysis; Visium HD and Xenium were each timed once.

bioRxiv preprint doi: https://doi.org/10.64898/2026.10.26.761912; this version posted October 30, 2026. The copyright holder for this preprint (which was not certified by peer review) is the author/funder, who has granted bioRxiv a license to display the preprint in perpetuity. It is made available under a CC-BY 4.0 International license.

827 # Supplementary Figures

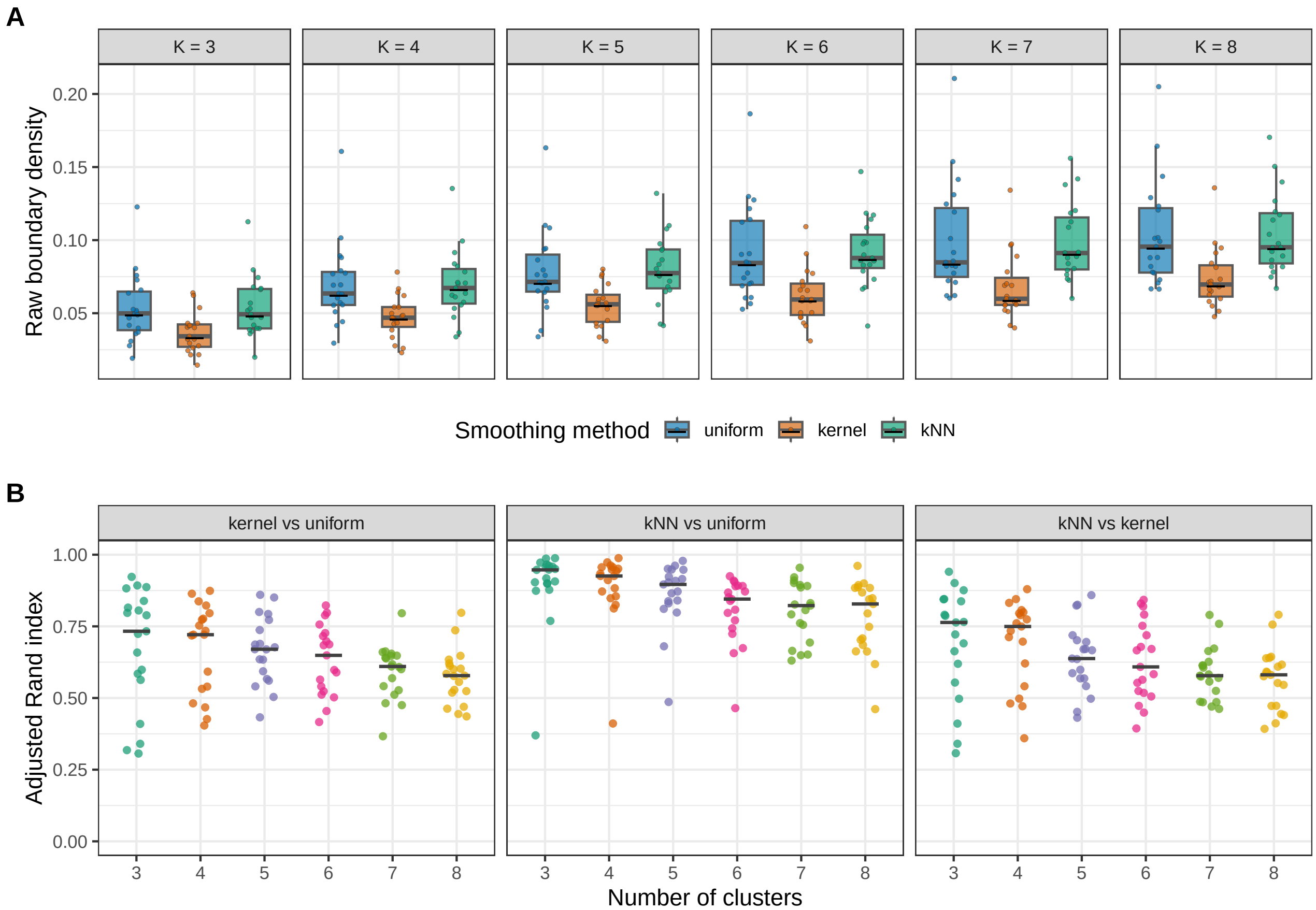

Supplementary Figure S1: GBM smoothing method sensitivity analysis. (A) Raw BD for smoothclust labels generated using uniform-radius, kernel, and k-nearest-neighbor smoothing across $K = 3, \dots, 8$. Boxes show medians and interquartile ranges, with whiskers extending to observations within 1.5 interquartile ranges; points represent individual GBM/glioma tissue sections and horizontal bars indicate medians. (B) Pairwise adjusted Rand index (ARI) between labelings from the three smoothing methods, grouped by cluster number and faceted by smoothing-method pair. Points represent individual sections and bars indicate medians. The BD metric was calculated using $k = 6$; uniform and kernel smoothing used $b = 0.05$, and k-nearest-neighbor smoothing used $k_s = 18$. In these settings, $h = bR$ is a hard radius for uniform smoothing and an exponential length scale with wider truncated support for kernel smoothing, whereas the k-nearest-neighbor setting fixes neighbor count.

bioRxiv preprint doi: https://doi.org/10.64898/2026.10.26.761912; this version posted October 30, 2026. The copyright holder for this preprint (which was not certified by peer review) is the author/funder, who has granted bioRxiv a license to display the preprint in perpetuity. It is made available under a CC-BY 4.0 International license.

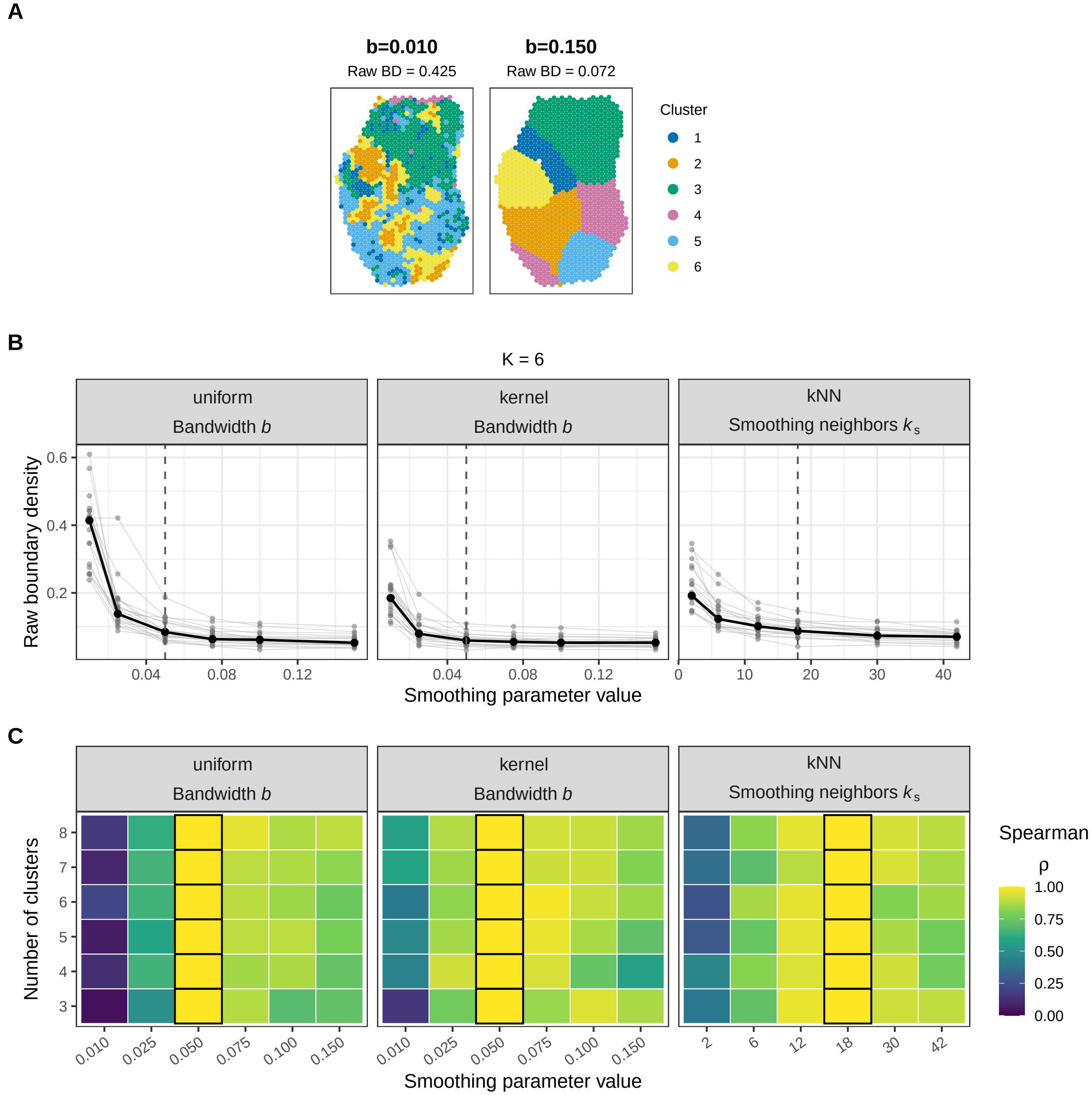

Supplementary Figure S2: GBM smoothing parameter sensitivity analysis. (A) Representative domain maps for section zh1019t1 with $K = 6$ using uniform smoothing at smaller bandwidth $b = 0.010$ (raw BD = 0.425; left) and larger bandwidth $b = 0.150$ (raw BD = 0.072; right). (B) Raw BD as a function of smoothing bandwidth or smoothing-neighbor count at $K = 6$. Gray lines represent sections and black points/lines indicate medians. Dashed vertical lines mark defaults. Uniform-smoothing bandwidth is expressed relative to each section's larger post-filtering coordinate range. (C) Spearman rank correlation between sample-level raw BD values at each setting and the corresponding default across $K = 3, \ldots, 8$. Black outlines mark default settings, whose correlations equal 1 by self-comparison. Defaults were $b = 0.05$ for uniform and kernel smoothing and $k_s = 18$ for nearest-neighbor smoothing. The BD metric was calculated using $k = 6$.

bioRxiv preprint doi: https://doi.org/10.64898/2026.10.26.761912; this version posted October 30, 2026. The copyright holder for this preprint (which was not certified by peer review) is the author/funder, who has granted bioRxiv a license to display the preprint in perpetuity. It is made available under a CC-BY 4.0 International license.

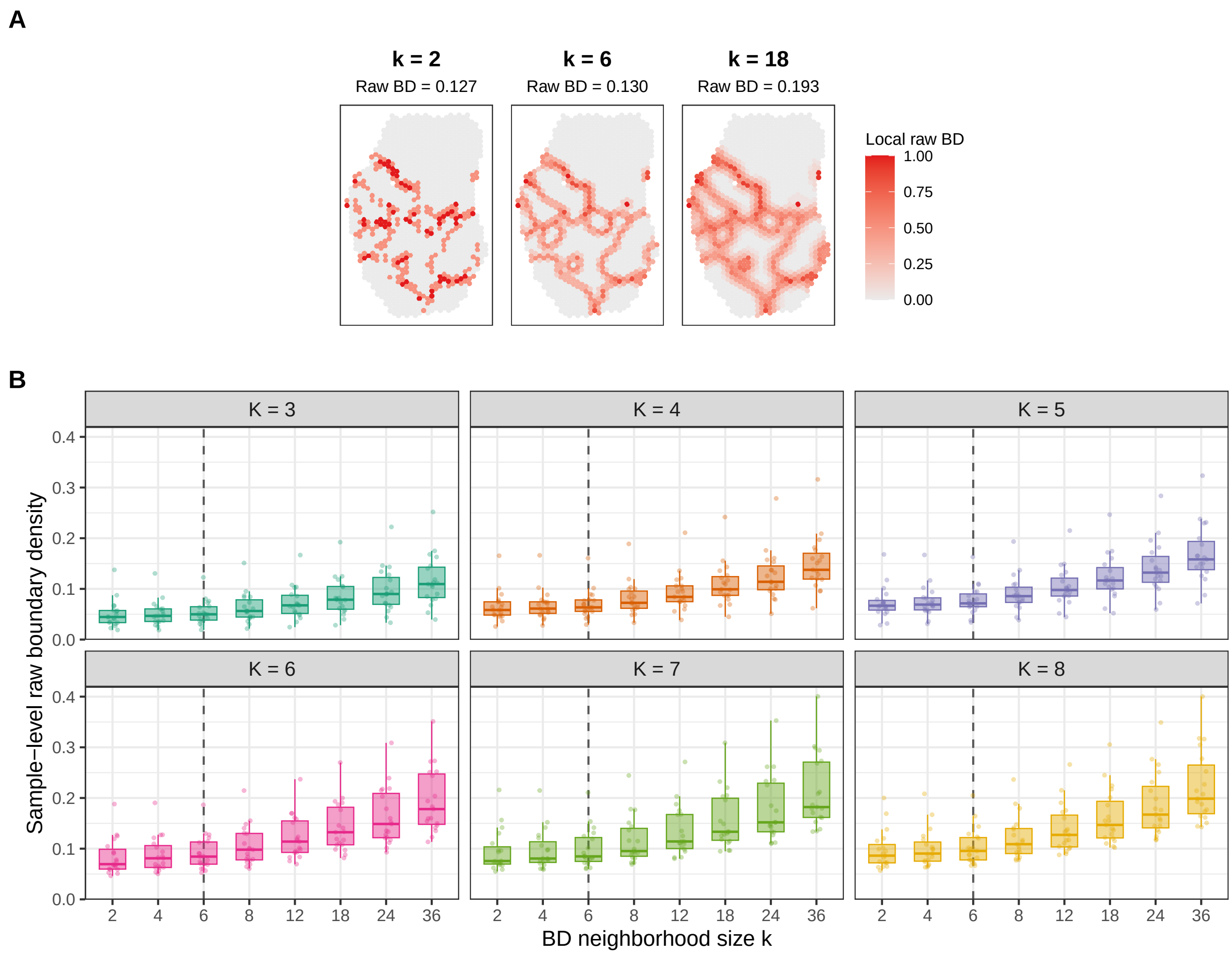

Supplementary Figure S3: BD metric neighborhood-size sensitivity in the GBM cohort. (A) Representative local raw BD maps for section zh1019t1 with $K = 6$, calculated using small ($k = 2$), default ($k = 6$), and large ($k = 18$) neighborhoods, from left to right. Values below each heading give sample-level raw BD. (B) Sample-level raw BD distributions across neighborhood sizes $k = 2, 4, 6, 8, 12, 18, 24, 36$, faceted by cluster number $K = 3, \ldots, 8$. Points represent the 19 sections; boxes show medians and interquartile ranges. Dashed vertical lines mark the default $k = 6$. smoothclust labels were held fixed.

bioRxiv preprint doi: https://doi.org/10.64898/2026.10.26.761912; this version posted October 30, 2026. The copyright holder for this preprint (which was not certified by peer review) is the author/funder, who has granted bioRxiv a license to display the preprint in perpetuity. It is made available under a CC-BY 4.0 International license.

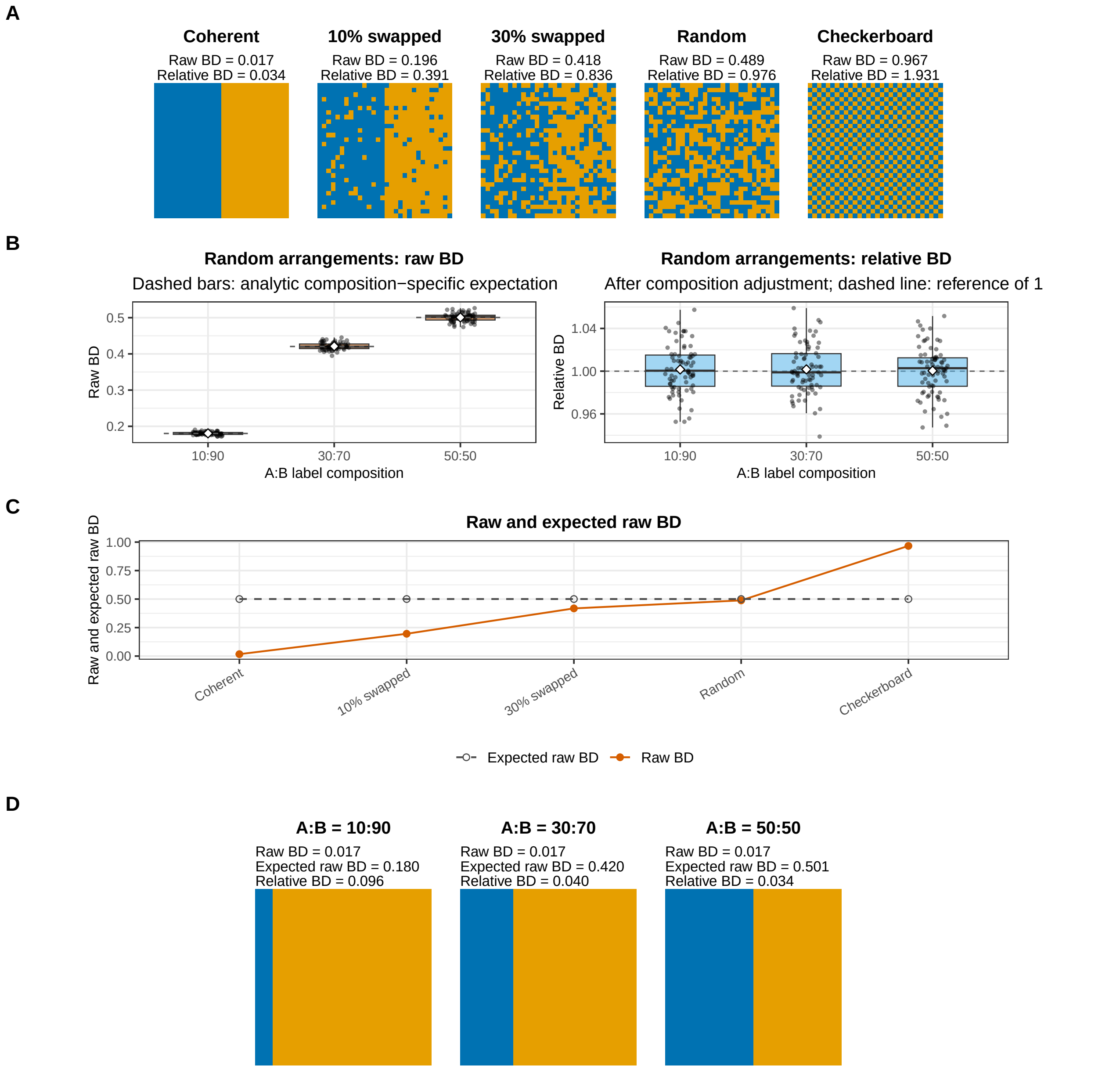

Supplementary Figure S4: Illustrating the composition adjustment underlying relative BD. (A) Two-label configurations on a 30 × 30 regular grid, each with exactly 450 locations per label. In panels A and D, label A is blue and label B is orange. (B) Raw and relative BD across 75 independent random arrangements at each exact label composition. Dashed bars mark analytically expected raw BD, and the dashed line marks the relative-BD reference of 1. Diamonds mark means. (C) Observed and analytically expected raw BD for the fixed-450:450 organization series. The random map is one realization; the checkerboard deliberately contains more label transitions than the random-label expectation. At fixed composition, raw and relative BD differ by a constant scaling; relative BD values are also shown in the map subtitles in panel A. (D) Straight-interface maps with 10:90, 30:70, and 50:50 compositions on the same grid, with raw BD, expected raw BD, and relative BD shown for each. The graph, interface orientation, and interface extent are fixed across these three maps, illustrating how approximately equal observed transition frequencies can yield different relative BD because the composition-specific references differ. All BD calculations used directed four-nearest-neighbor graphs; expected raw BD was calculated analytically.

bioRxiv preprint doi: https://doi.org/10.64898/2026.10.26.761912; this version posted October 30, 2026. The copyright holder for this preprint (which was not certified by peer review) is the author/funder, who has granted bioRxiv a license to display the preprint in perpetuity. It is made available under a CC-BY 4.0 International license.

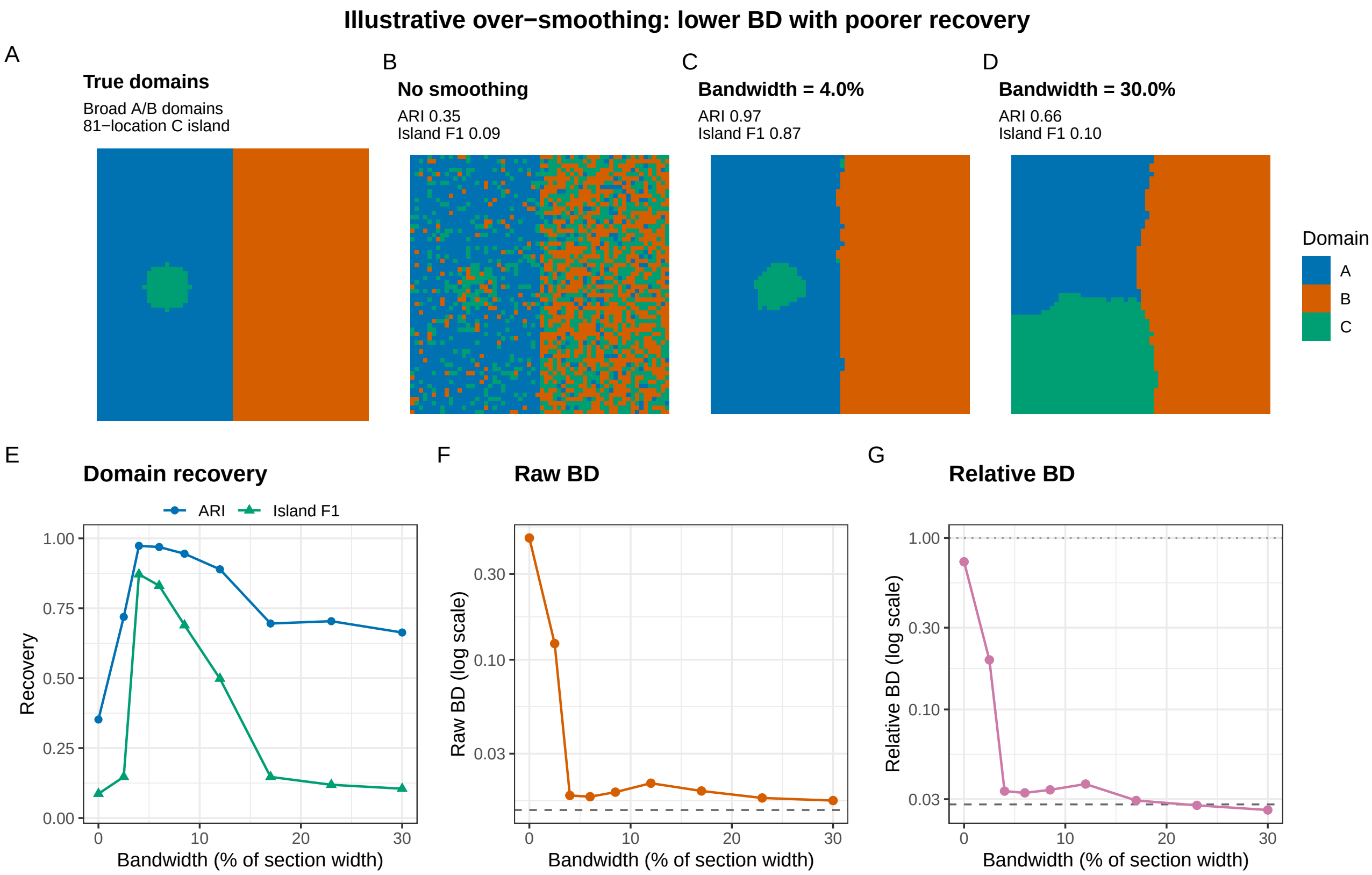

Supplementary Figure S5: Illustrative over-smoothing simulation with lower BD metric values and poorer domain recovery. (A) True labels for a three-domain count simulation on a 60×60 grid with broad left and right domains and an 81-location circular third-domain island. (B–D) Inferred three-cluster maps without smoothing, at the 4% uniform bandwidth (highest ARI and island F1 in this realization), and at the largest evaluated bandwidth of 30% of the coordinate range. Cluster identities were aligned to truth only for display. (E) ARI and island F1 across all evaluated bandwidths. (F) Raw BD and (G) relative BD, the composition-adjusted summary, for the inferred labels; dashed lines mark the values for the true labels and the dotted line in panel G marks the fixed-count random-label reference. Both F and G use logarithmic y-axes. The feature set, number of principal components (10), $K = 3$, coordinates, and directed four-nearest-neighbor settings were held fixed; PCA was recomputed for each expression matrix. This analysis used one illustrative count realization.

bioRxiv preprint doi: https://doi.org/10.64898/2026.10.26.761912; this version posted October 30, 2026. The copyright holder for this preprint (which was not certified by peer review) is the author/funder, who has granted bioRxiv a license to display the preprint in perpetuity. It is made available under a CC-BY 4.0 International license.

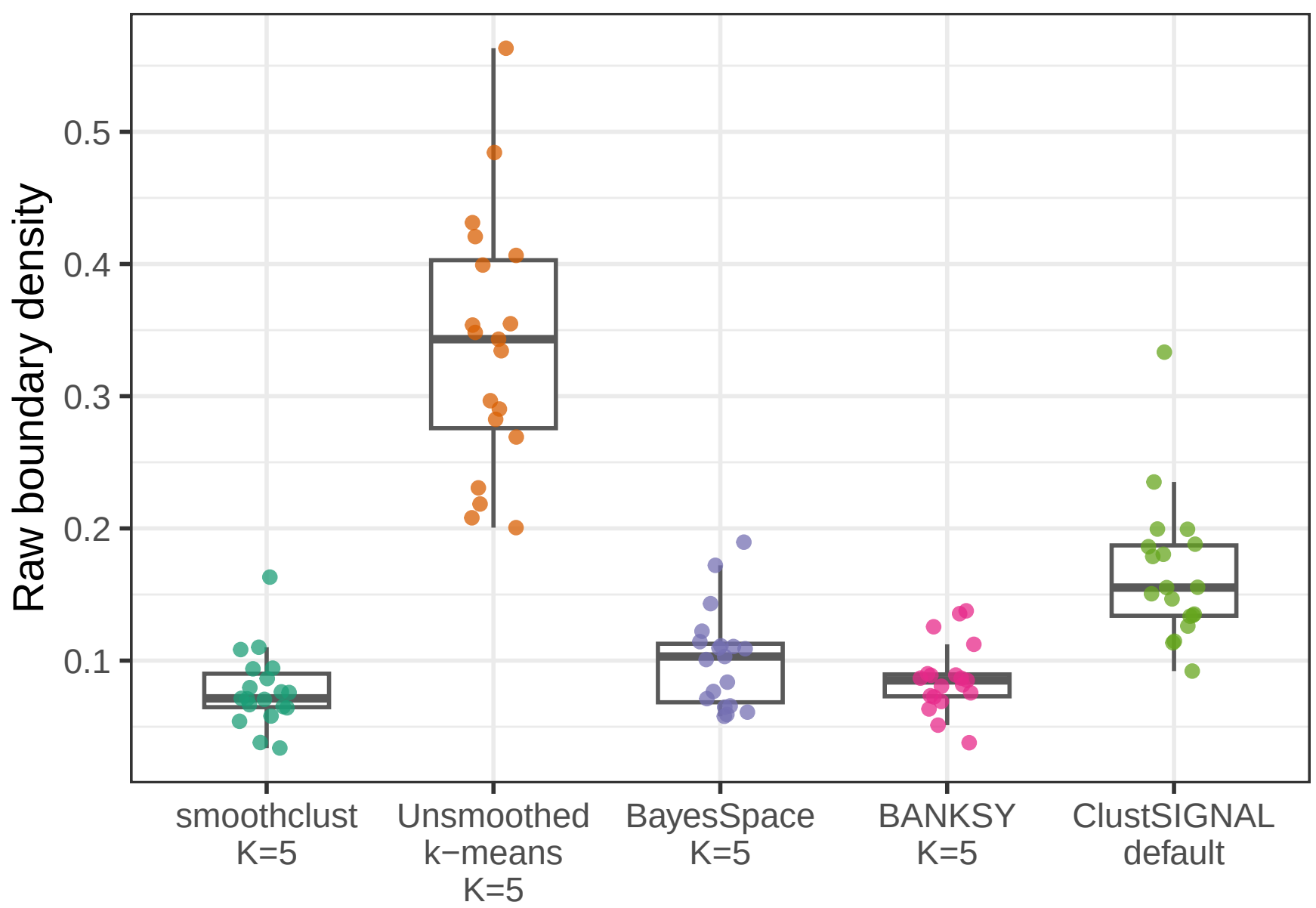

Supplementary Figure S6: Sample-level raw BD for labels generated by different clustering methods. Raw BD for smoothclust, the matched unsmoothed k-means baseline, and the external clustering algorithms BayesSpace, BANKSY, and ClustSIGNAL across 19 GBM/glioma sections. Boxes show medians and interquartile ranges, with whiskers extending to observations within 1.5 interquartile ranges; points represent individual sections. The first four methods are shown at $K = 5$; ClustSIGNAL used default parameters and returned 3–10 labels. All BD calculations used $k = 6$.

bioRxiv preprint doi: https://doi.org/10.64898/2026.10.26.761912; this version posted October 30, 2026. The copyright holder for this preprint (which was not certified by peer review) is the author/funder, who has granted bioRxiv a license to display the preprint in perpetuity. It is made available under a CC-BY 4.0 International license.

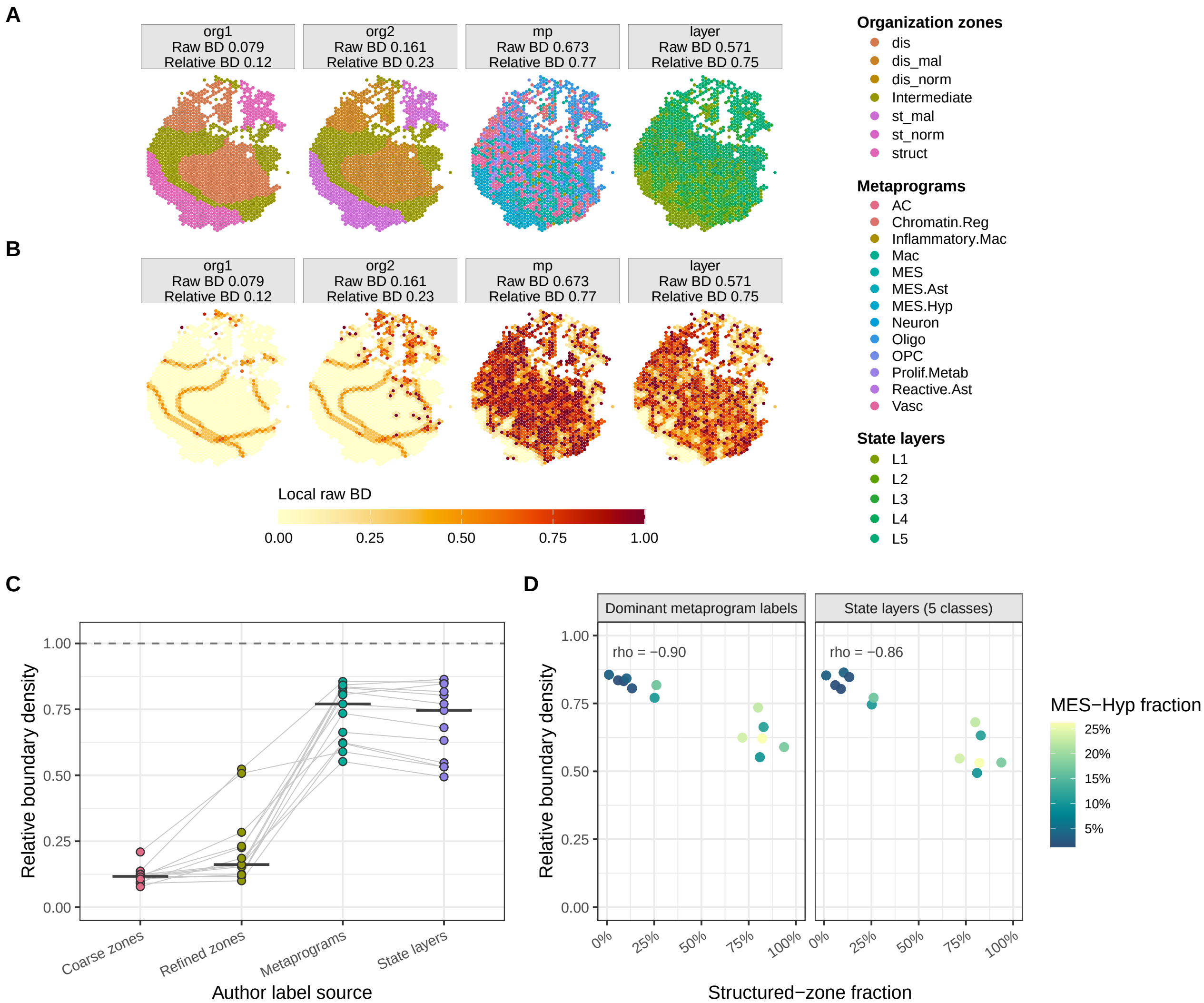

Supplementary Figure S7: BD summarizes published GBM tissue annotations. (A) Representative Greenwald organization zones (`org1` and `org2`), dominant metaprograms (`mp`), and deposited state-layer annotations (`layer`) in section zh1019inf. Chromatin-Reg-dominant spots retained their deposited L1 assignments. (B) Local raw BD maps; facet labels report sample-level raw and relative BD. (C) Relative BD across the 13 annotated sections for Coarse zones (`org1`), Refined zones (`org2`), Metaprograms (`mp`), and State layers (`layer`); lines connect fields from the same section. Median raw BD was 0.049 for `org1`, 0.071 for `org2`, 0.673 for `mp`, and 0.571 for `layer`. The dashed line marks relative BD = 1. (D) Relative BD for `mp` and `layer` against structured-zone fraction; color indicates MES-Hyp fraction. Correlations are descriptive across 13 sections from five tumors. All BD calculations used $k = 6$.

bioRxiv preprint doi: https://doi.org/10.64898/2026.10.26.761912; this version posted October 30, 2026. The copyright holder for this preprint (which was not certified by peer review) is the author/funder, who has granted bioRxiv a license to display the preprint in perpetuity. It is made available under a CC-BY 4.0 International license.

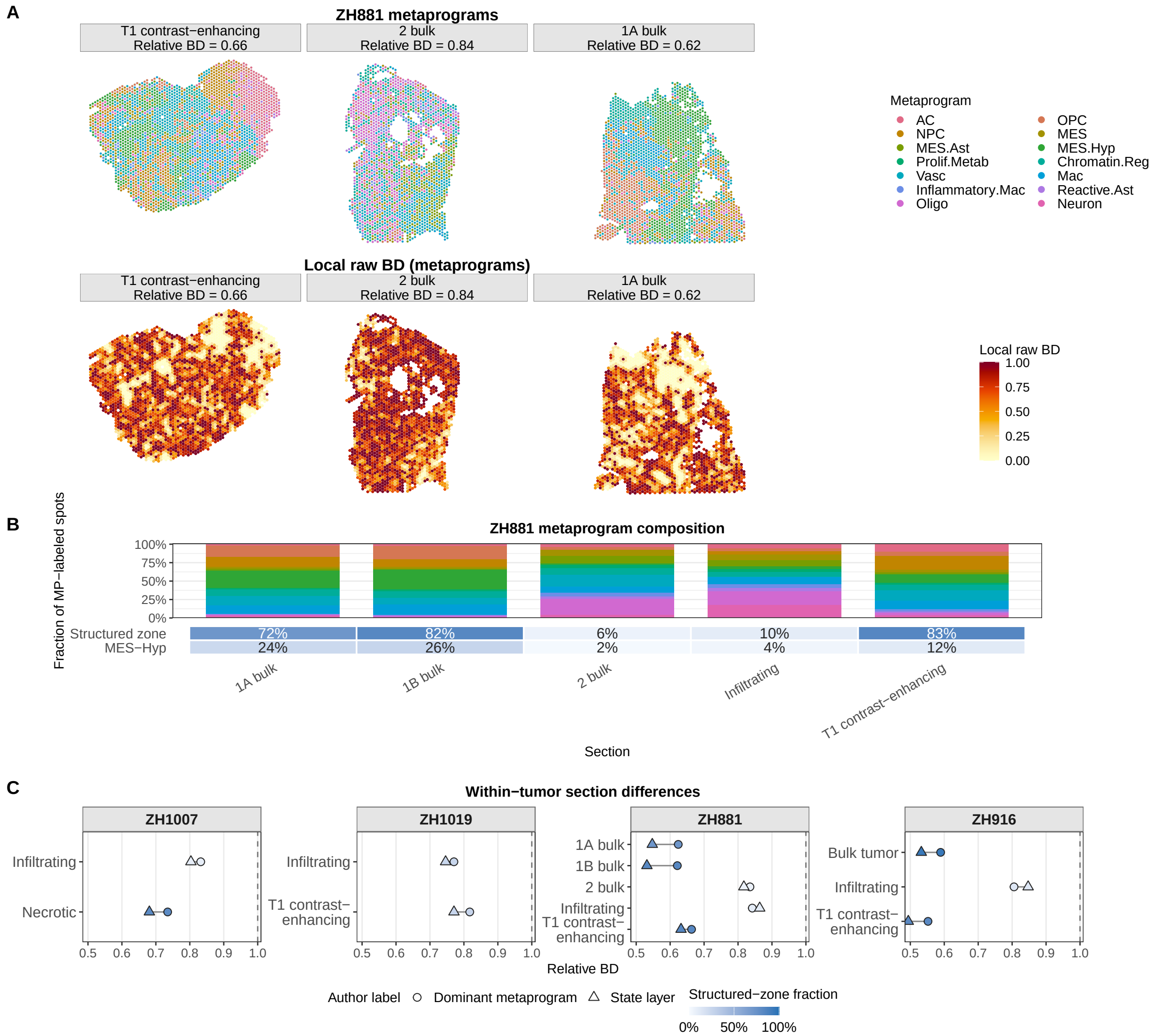

Supplementary Figure S8: BD summarizes variation among regions within GBM tumors. (A) Dominant-metaprogram label maps and corresponding local raw BD maps for three ZH881 sections; headings report metaprogram relative BD. (B) Metaprogram composition across all five ZH881 sections, with structured-zone and MES-Hyp percentages below the composition bars. (C) Metaprogram and state-layer relative BD for 12 regional sections from four tumors; fill indicates structured-zone fraction and lines connect label sources from the same section. Dashed vertical lines mark relative BD = 1. State-layer values retain the deposited L1 assignments for Chromatin-Reg-dominant spots. BD was calculated on labeled-spot $k = 6$ graphs using the analytic fixed-count reference.

bioRxiv preprint doi: https://doi.org/10.64898/2026.10.26.761912; this version posted October 30, 2026. The copyright holder for this preprint (which was not certified by peer review) is the author/funder, who has granted bioRxiv a license to display the preprint in perpetuity. It is made available under a CC-BY 4.0 International license.

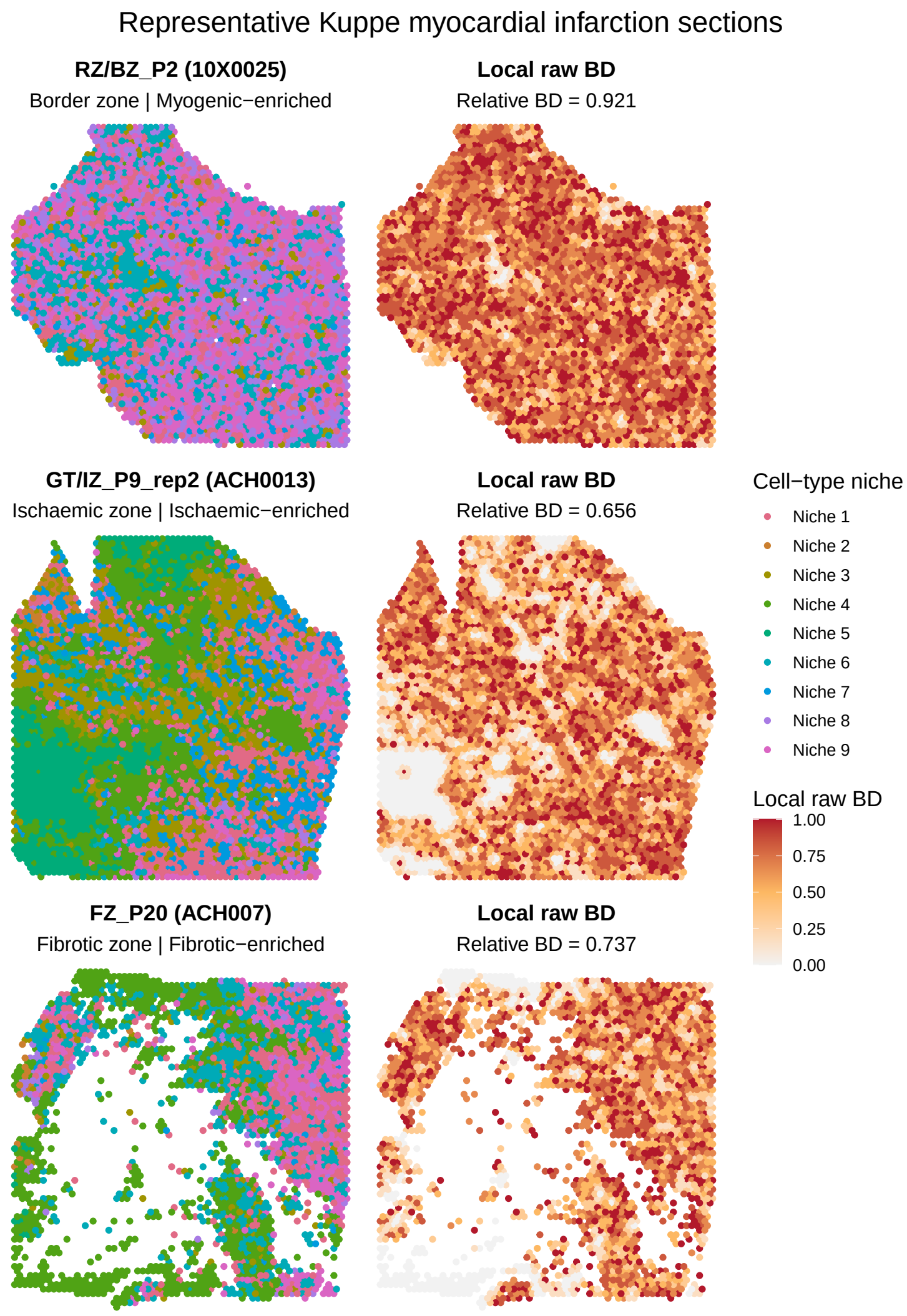

Supplementary Figure S9: Published cell-type niches and local raw BD in representative myocardial sections. For each author-defined tissue category, we selected the section closest to its median relative BD: RZ/BZ_P2 (myogenic-enriched; 0.921), GT/IZ_P9_rep2 (ischaemic-enriched; 0.656), and FZ_P20 (fibrotic-enriched; 0.737). Left panels show the nine published computational cell-type-niche labels; right panels show corresponding local raw BD, the fraction of each spot's six nearest neighbors assigned to a different label.

bioRxiv preprint doi: https://doi.org/10.64898/2026.10.26.761912; this version posted October 30, 2026. The copyright holder for this preprint (which was not certified by peer review) is the author/funder, who has granted bioRxiv a license to display the preprint in perpetuity. It is made available under a CC-BY 4.0 International license.

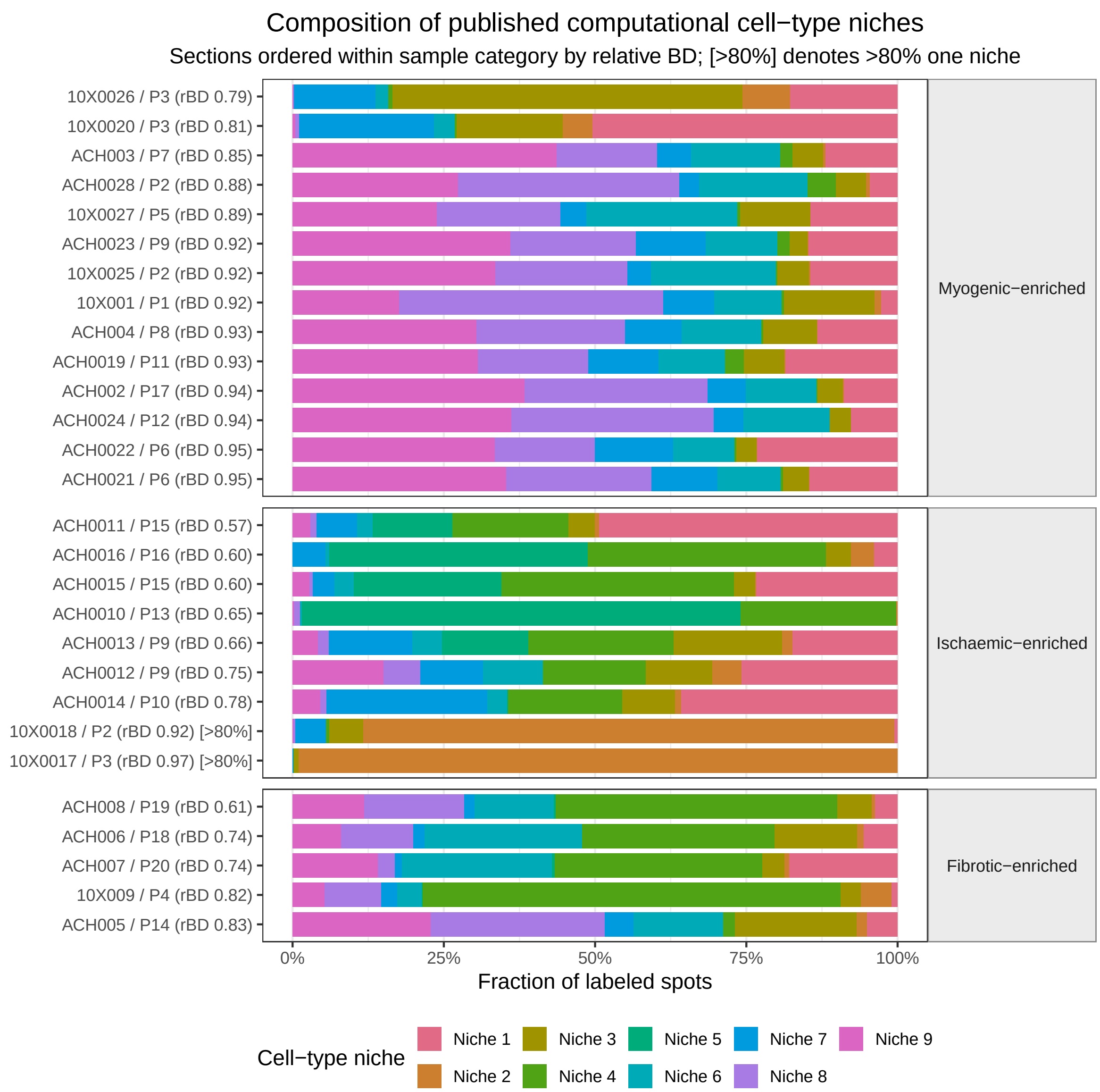

Supplementary Figure S10: Composition of published myocardial cell-type niches across 28 sections. Fraction of spots assigned to each of the nine computational cell-type niches. Sections are grouped by the author-defined tissue category and ordered within each category by relative BD. Labels identify sections and patients and report relative BD; [> 80%] marks sections in which one niche occupies more than 80% of spots.

bioRxiv preprint doi: https://doi.org/10.64898/2026.10.26.761912; this version posted October 30, 2026. The copyright holder for this preprint (which was not certified by peer review) is the author/funder, who has granted bioRxiv a license to display the preprint in perpetuity. It is made available under a CC-BY 4.0 International license.

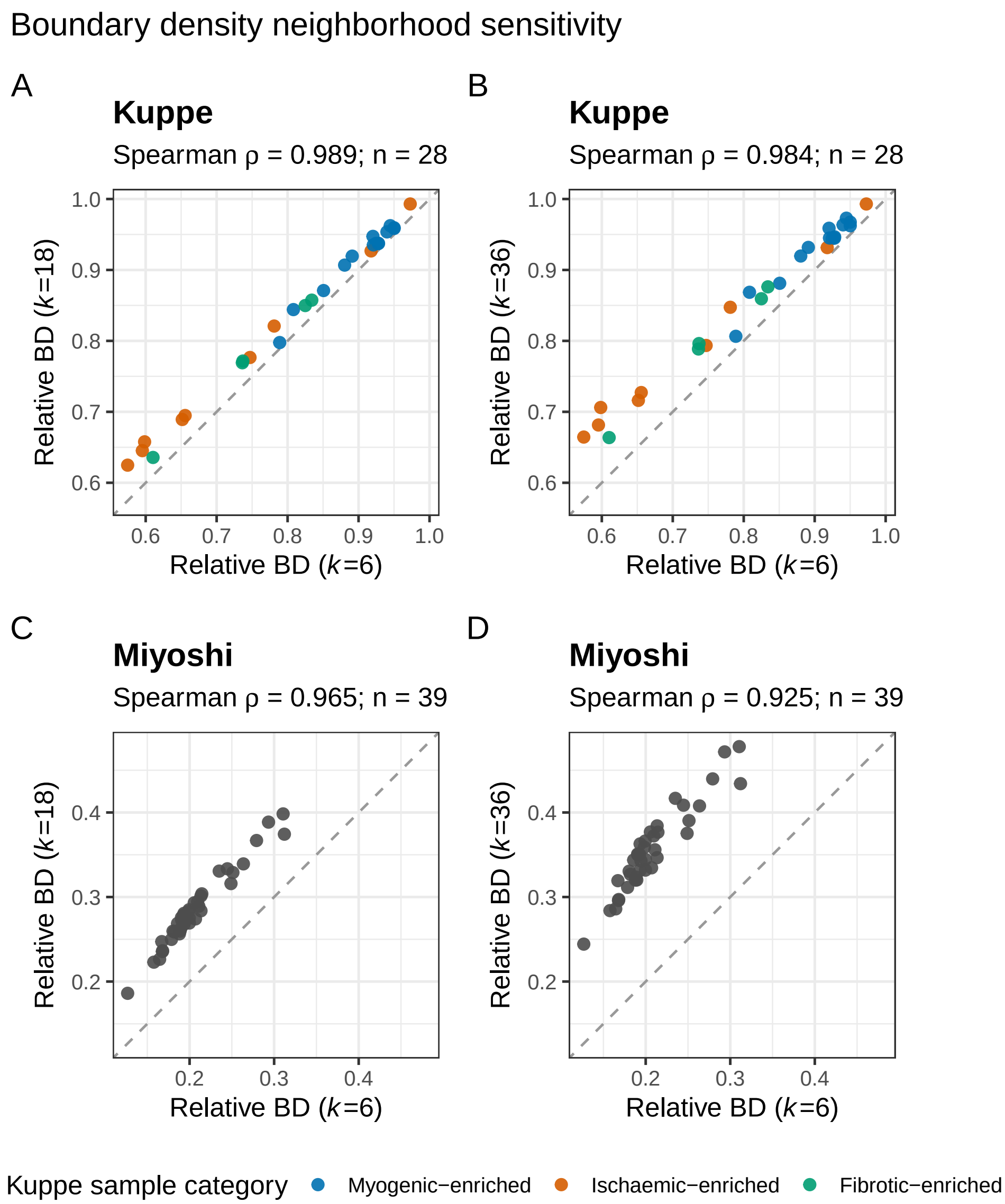

Supplementary Figure S11: BD neighborhood-size sensitivity for published myocardial and cortical annotations. (A–B) Relative BD at $k = 18$ and $k = 36$, respectively, against $k = 6$ for the nine published Kuppe cell-type-niche labels across 28 sections from 20 patients; colors denote the author-defined tissue categories. (C–D) The corresponding comparisons for the original nine-region BayesSpace $q = 9$ annotation across 39 Miyoshi frontal-cortex sections. Each point is one section; dashed lines indicate equality. Axes have equal scaling, with common limits within each dataset's row, and panels report Spearman correlations. For $k = 18$ and $k = 36$, respectively, median (maximum) absolute changes in relative BD were 0.025 (0.059) and 0.039 (0.108) for Kuppe, and 0.078 (0.096) and 0.145 (0.182) for Miyoshi. Section rankings remained strongly correlated while absolute values changed, particularly for Miyoshi.

bioRxiv preprint doi: https://doi.org/10.64898/2026.10.26.761912; this version posted October 30, 2026. The copyright holder for this preprint (which was not certified by peer review) is the author/funder, who has granted bioRxiv a license to display the preprint in perpetuity. It is made available under a CC-BY 4.0 International license.

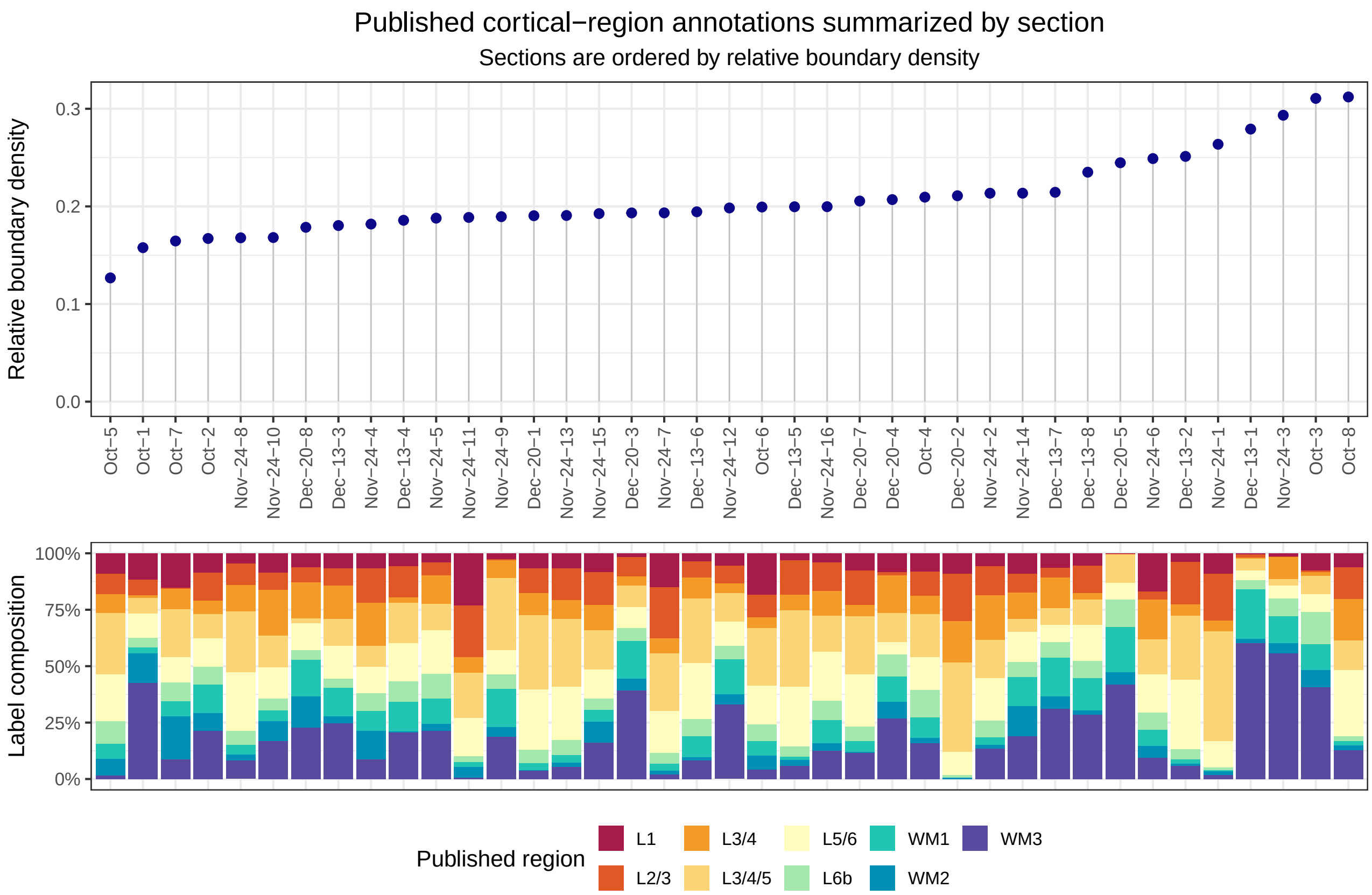

Supplementary Figure S12: Relative BD and label composition across Miyoshi frontal-cortex sections. Top: sample-level relative BD for the published nine-region BayesSpace annotation across 39 sections, ordered by increasing value. Bottom: fractions of spots assigned to each region in the same section order. Section identifiers are abbreviated by omitting 2021 and Human/VisiumHuman and replacing underscores with hyphens. BD was calculated using $k = 6$ on the transformed Visium lattice.

bioRxiv preprint doi: https://doi.org/10.64898/2026.10.26.761912; this version posted October 30, 2026. The copyright holder for this preprint (which was not certified by peer review) is the author/funder, who has granted bioRxiv a license to display the preprint in perpetuity. It is made available under a CC-BY 4.0 International license.

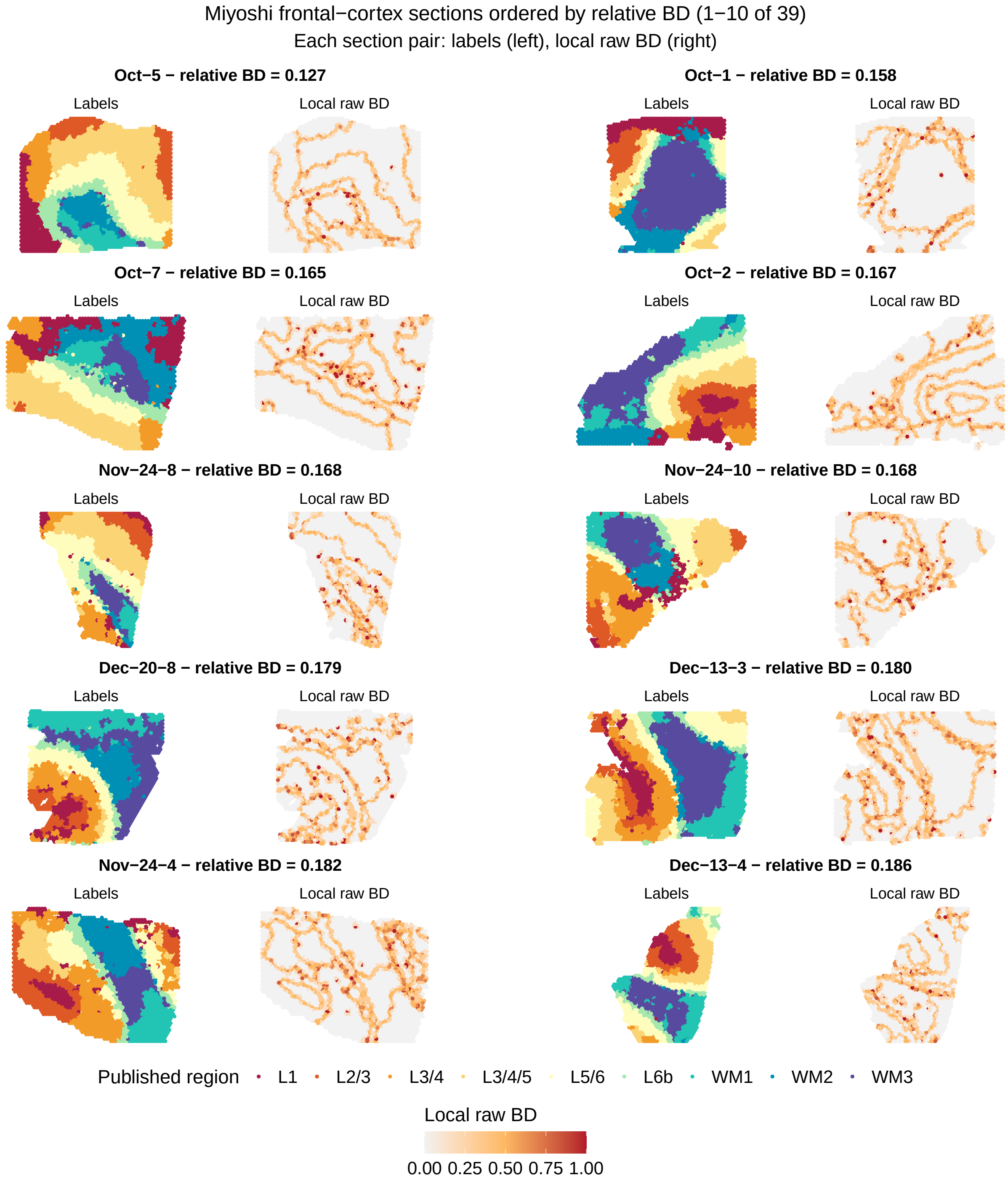

Supplementary Figure S13: Published cortical-region annotations and local raw BD across all 39 Miyoshi frontal-cortex sections. Sections 1–10 of 39, ordered by sample-level relative BD for the published nine-region BayesSpace annotation. Read each row from left to right, with up to two section-pairs per row separated by a gutter. Each shared heading gives an abbreviated section identifier (omitting 2021 and Human/VisiumHuman) and sample-level relative BD. For each section, the left map shows the $q = 9$ annotation and the right map shows corresponding local raw BD calculated using $k = 6$. Each page has one annotation legend and one common 0–1 local raw-BD legend. These spatially informed labels provide an annotation-summary example.

bioRxiv preprint doi: https://doi.org/10.64898/2026.10.26.761912; this version posted October 30, 2026. The copyright holder for this preprint (which was not certified by peer review) is the author/funder, who has granted bioRxiv a license to display the preprint in perpetuity. It is made available under a CC-BY 4.0 International license.

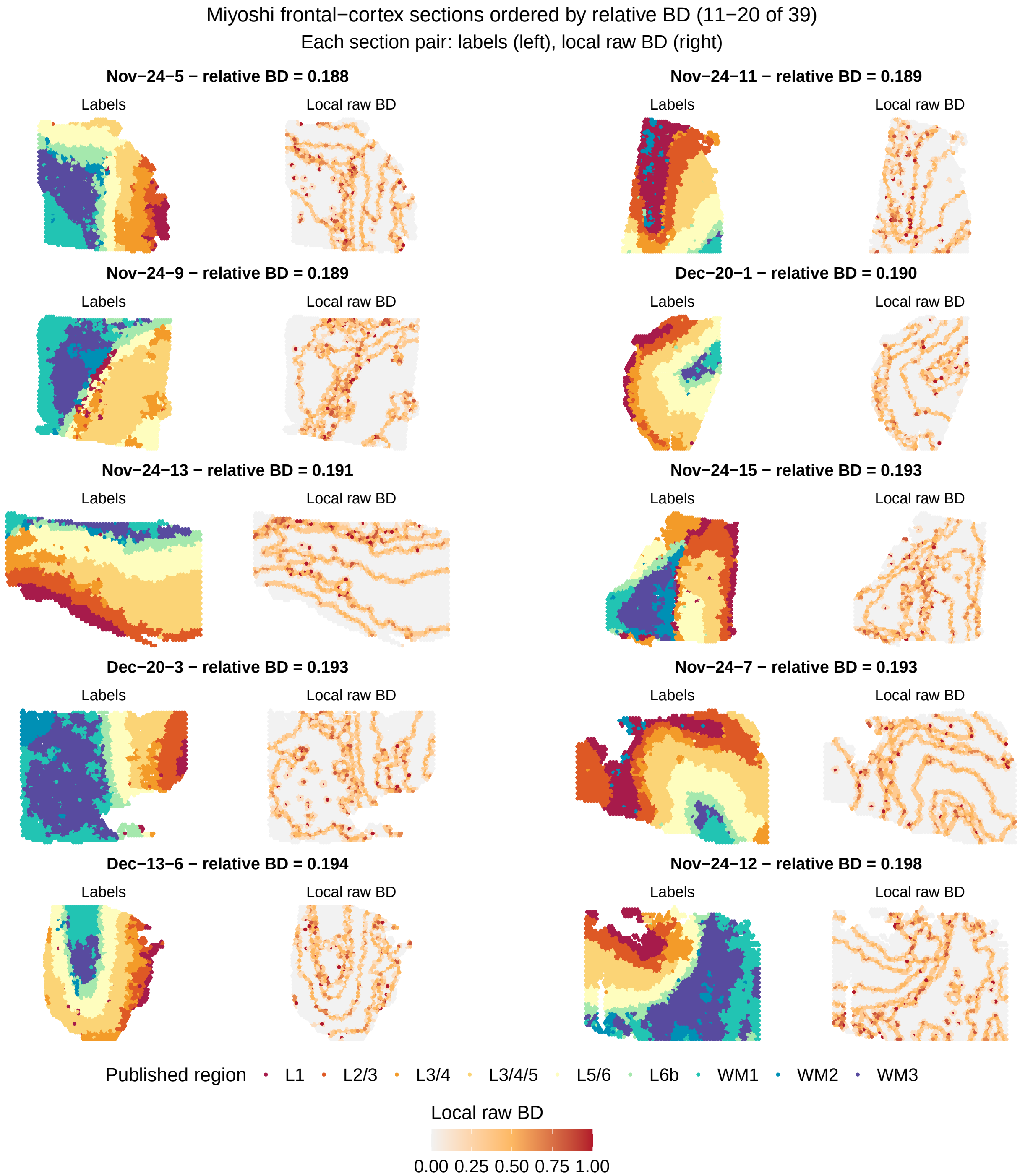

Supplementary Figure S13: Published cortical-region annotations and local raw BD across all 39 Miyoshi frontal-cortex sections (continued). Sections 11–20 in relative-BD order.

bioRxiv preprint doi: https://doi.org/10.64898/2026.10.26.761912; this version posted October 30, 2026. The copyright holder for this preprint (which was not certified by peer review) is the author/funder, who has granted bioRxiv a license to display the preprint in perpetuity. It is made available under a CC-BY 4.0 International license.

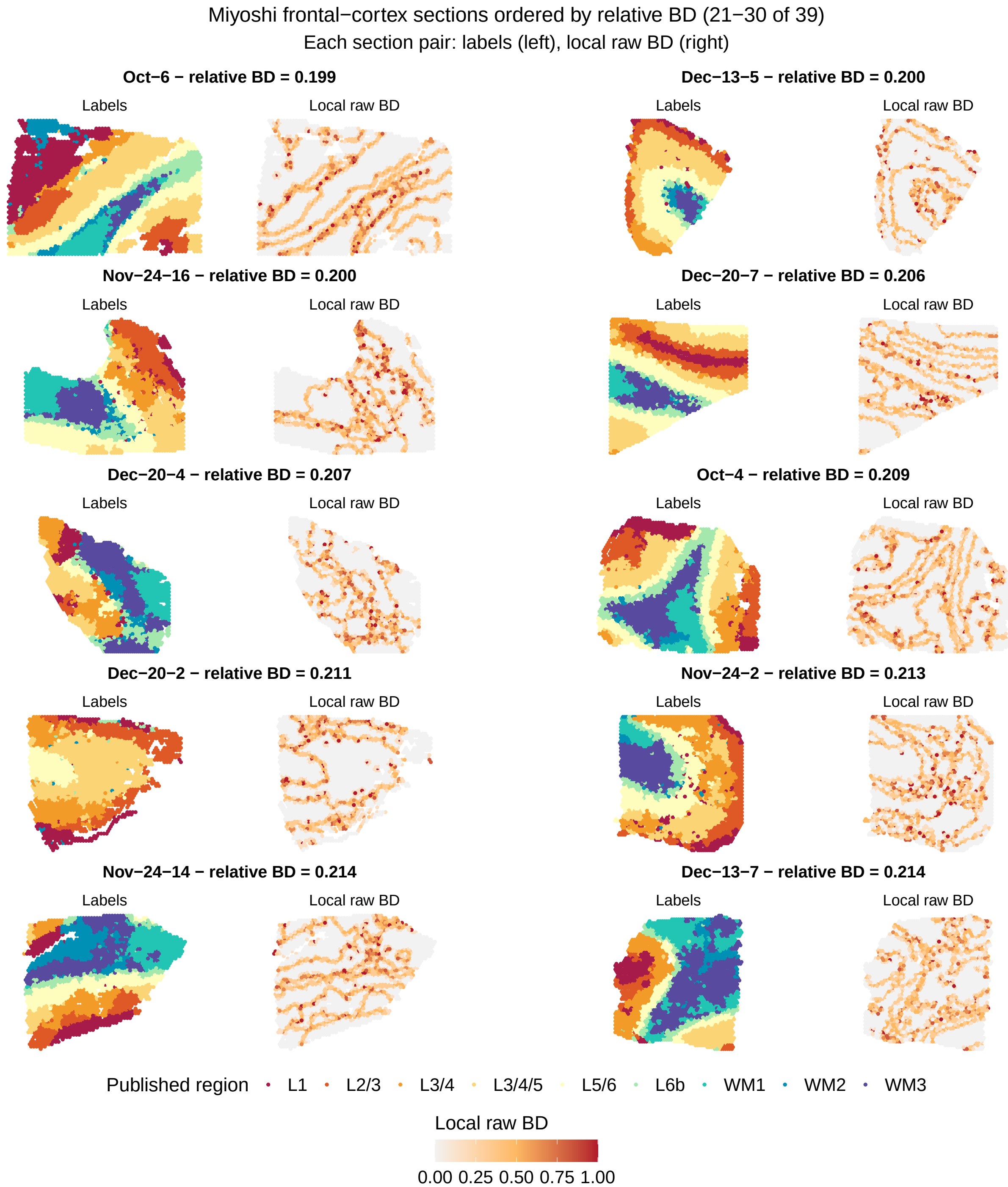

Supplementary Figure S13: Published cortical-region annotations and local raw BD across all 39 Miyoshi frontal-cortex sections (continued). Sections 21–30 in relative-BD order.

bioRxiv preprint doi: https://doi.org/10.64898/2026.10.26.761912; this version posted October 30, 2026. The copyright holder for this preprint (which was not certified by peer review) is the author/funder, who has granted bioRxiv a license to display the preprint in perpetuity. It is made available under a CC-BY 4.0 International license.

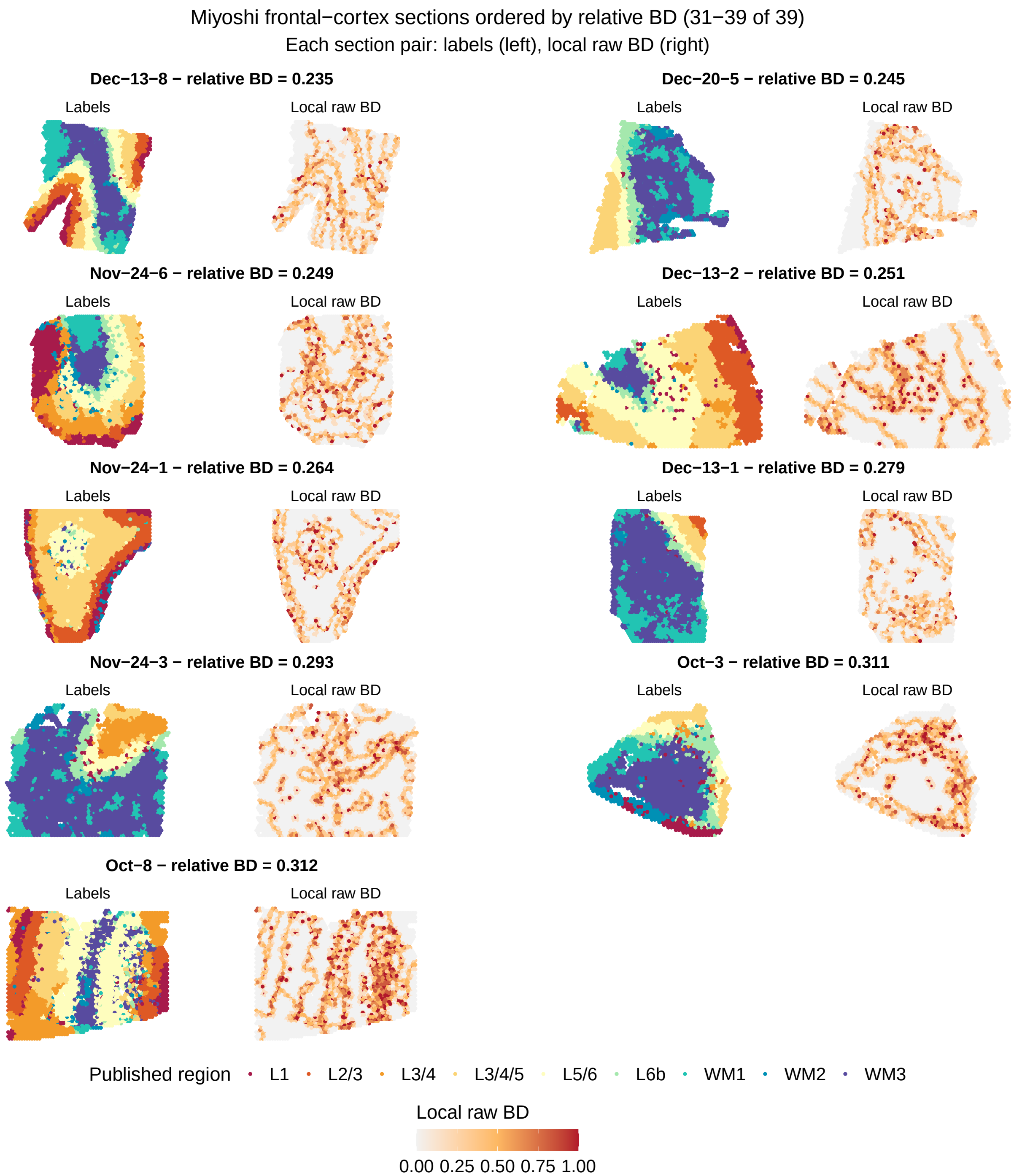

Supplementary Figure S13: Published cortical-region annotations and local raw BD across all 39 Miyoshi frontal-cortex sections (continued). Sections 31–39 in relative-BD order.

bioRxiv preprint doi: https://doi.org/10.64898/2026.10.26.761912; this version posted October 30, 2026. The copyright holder for this preprint (which was not certified by peer review) is the author/funder, who has granted bioRxiv a license to display the preprint in perpetuity. It is made available under a CC-BY 4.0 International license.

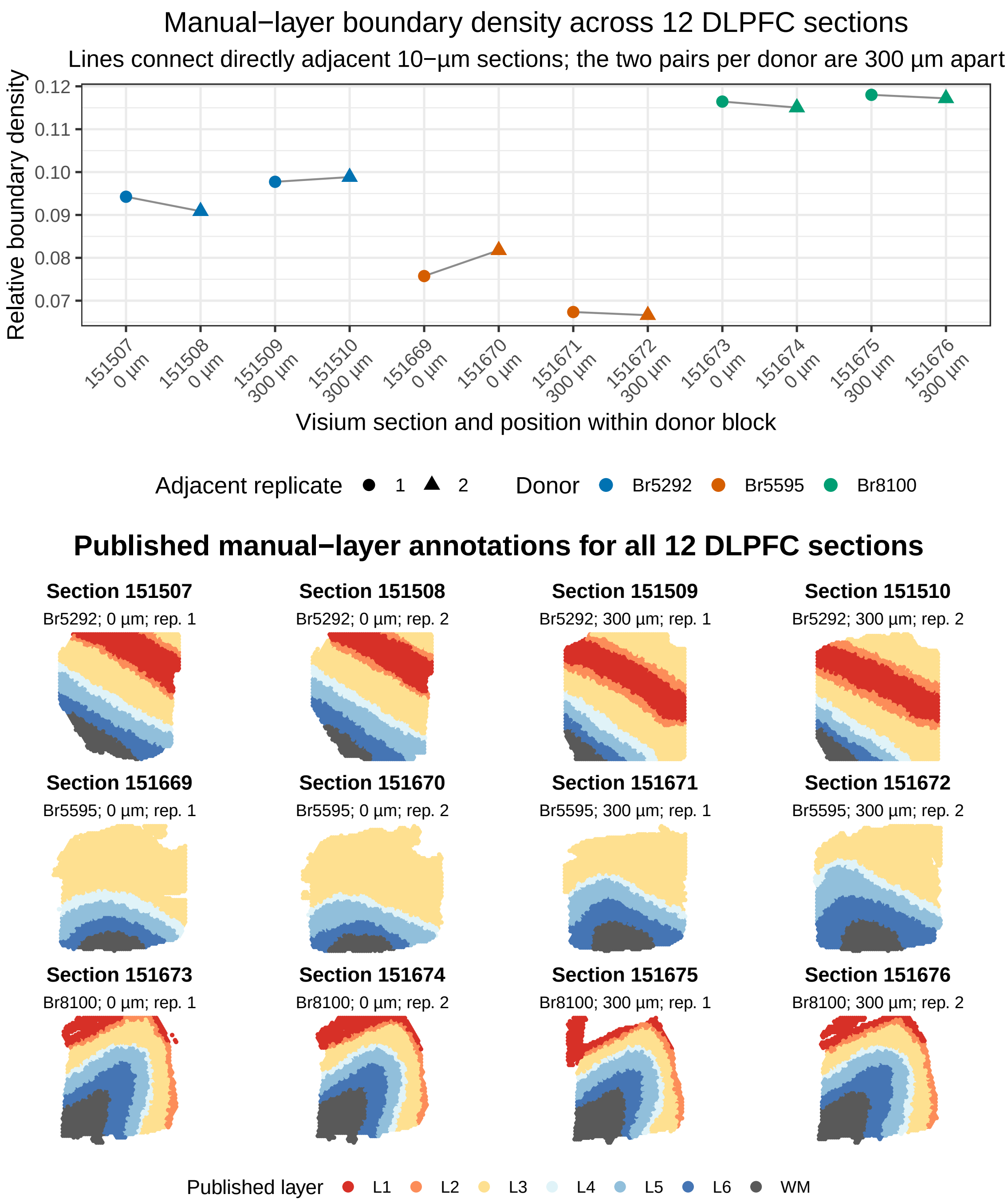

Supplementary Figure S14: Relative BD and published manual annotations across 12 Maynard DLPFC sections. Top: relative BD for sections from three donors. Color denotes donor, shape distinguishes replicate 1 from replicate 2, and lines connect directly adjacent 10 μm sections. Each donor contributed two adjacent pairs separated by 300 μm. The comparison is descriptive and does not treat sections as independent replicates. Bottom: manual annotation maps. Rows correspond to donors Br5292, Br5595, and Br8100. Within each row, the first two columns form the adjacent pair at position 0 and the last two columns form the adjacent pair at 300 μm in the tissue block. Colors denote layers L1–L6 and white matter. Br5595 lacked L1 and a distinct L2, and ambiguous L2/L3 spots were assigned to L3 in the original annotations; between-donor BD differences may therefore also reflect anatomical coverage and annotation granularity. The 352 spots without a manual layer assignment were excluded; all nonmissing manual labels were plotted, including 534 spots marked `discard=TRUE` in the deposited object.

bioRxiv preprint doi: https://doi.org/10.64898/2026.10.26.761912; this version posted October 30, 2026. The copyright holder for this preprint (which was not certified by peer review) is the author/funder, who has granted bioRxiv a license to display the preprint in perpetuity. It is made available under a CC-BY 4.0 International license.

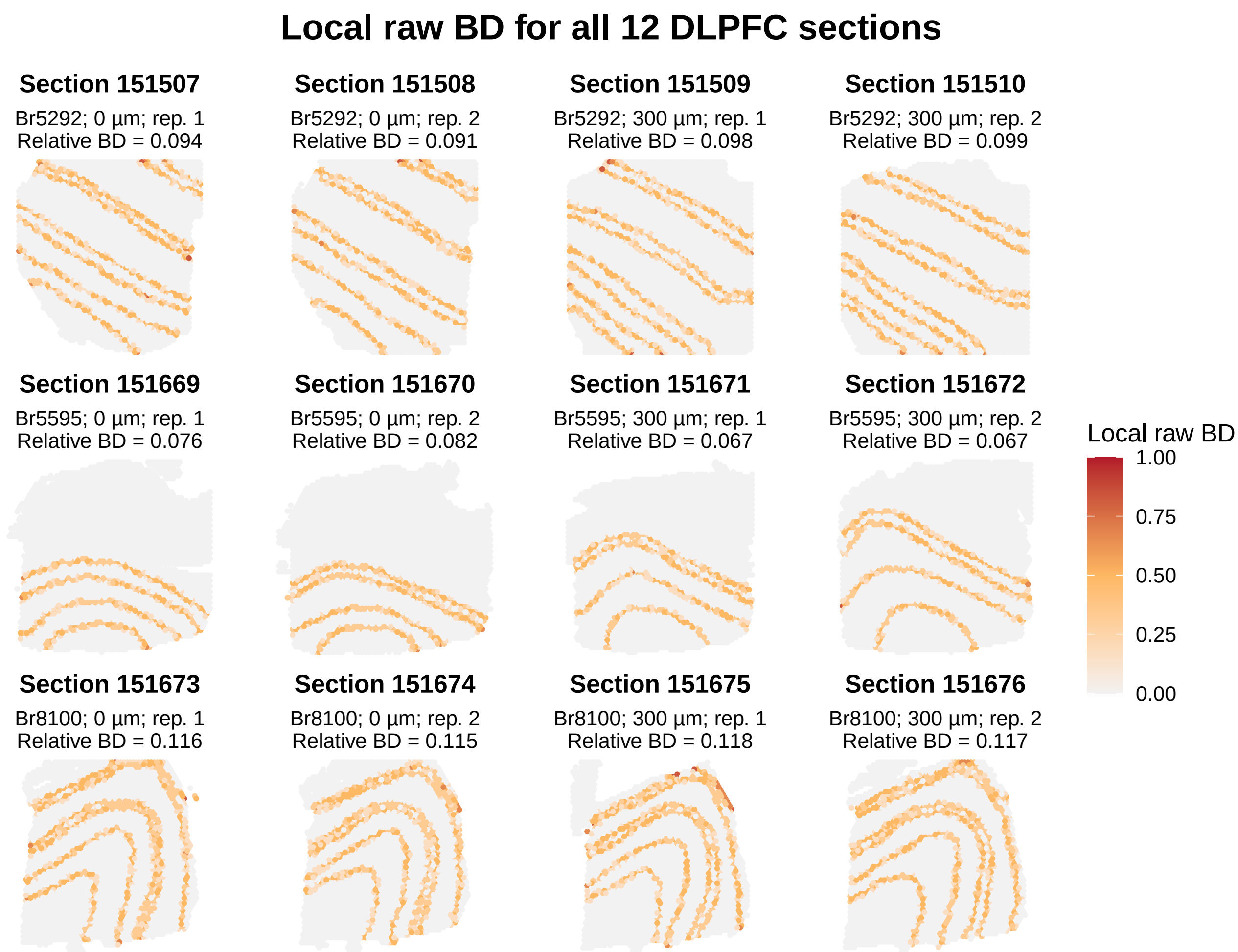

Supplementary Figure S14: Local raw BD for all 12 Maynard DLPFC sections (continued). Sections are shown in the same donor, position, and replicate order as on the preceding page. Locations are colored by the fraction of their six nearest neighbors assigned to a different published anatomical label (cortical layer or white matter), using a common 0–1 scale; subtitles report relative BD for each section.

bioRxiv preprint doi: https://doi.org/10.64898/2026.10.26.761912; this version posted October 30, 2026. The copyright holder for this preprint (which was not certified by peer review) is the author/funder, who has granted bioRxiv a license to display the preprint in perpetuity. It is made available under a CC-BY 4.0 International license.

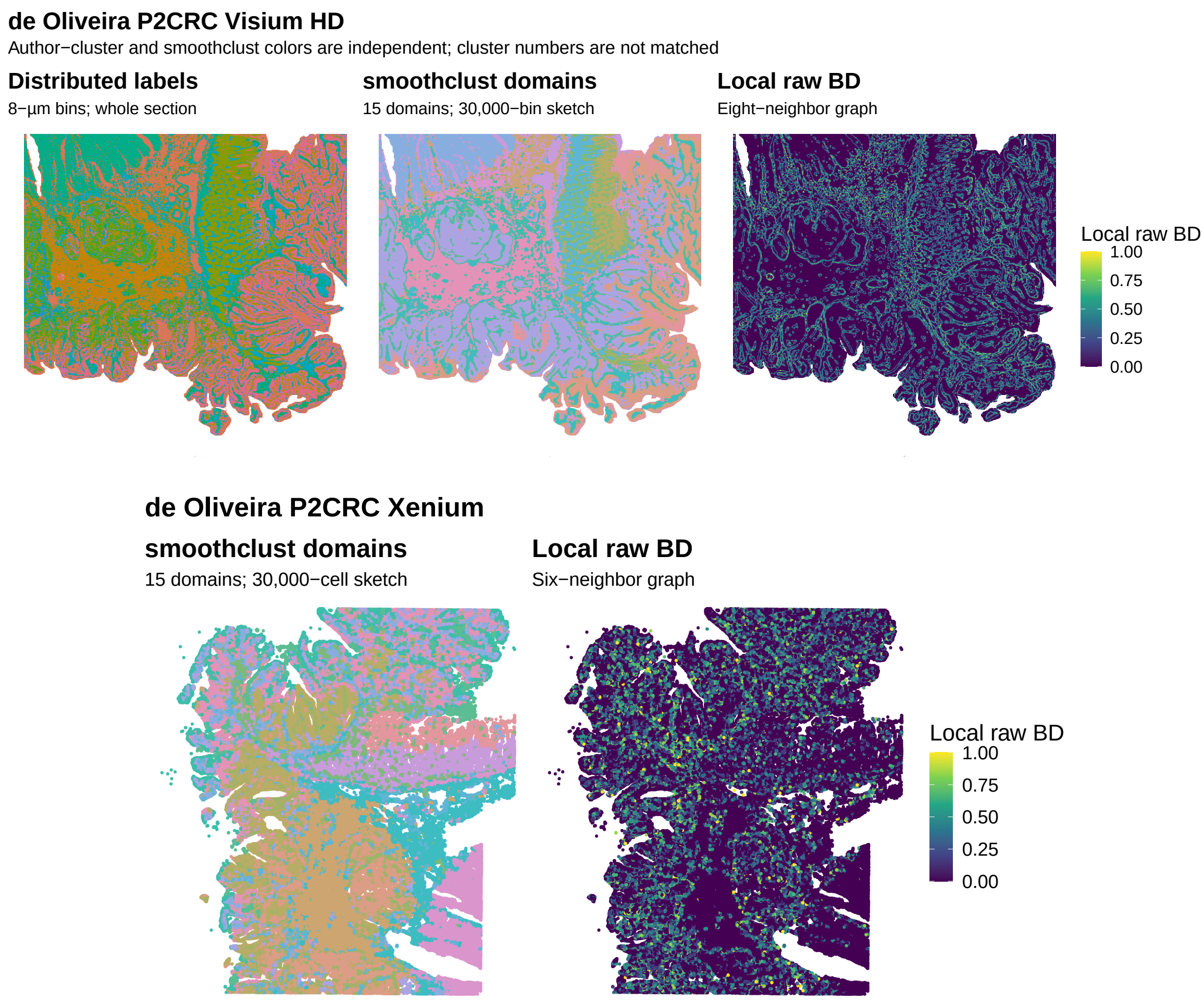

Boundary density within each illustrative analysis

Platform / label source	BD neighbors (k)	Raw BD	Expected raw BD	Relative BD	Locations
Visium HD / distributed labels	8	0.3263	0.8966	0.3639	545,850
Visium HD / smoothclust labels	8	0.1612	0.8917	0.1808	545,850
Xenium / smoothclust labels	6	0.1704	0.9180	0.1856	339,681

Visium HD: 8−μm bins; Xenium: segmented cells. Expected raw BD uses fixed label counts.
Each row describes one label map; values are not directly comparable across platforms or label sources.

Supplementary Figure S15: Illustrative full-section smoothclust and BD metric analyses on Visium HD and Xenium. Top: the complete P2CRC Visium HD section (545,850 nonempty 8 μm bins), showing 15 cluster labels distributed with the source object, 15 smoothclust-derived domains, and local raw BD for these domains using $k = 8$ spatial neighbors. Colors and numeric identifiers are independent between label maps and do not imply one-to-one correspondence. Middle: a serial Xenium section from the same P2CRC FFPE tissue block (339,681 nonempty segmented cells), showing 15 smoothclust-derived domains and local raw BD using $k = 6$ cell neighbors. Both workflows used $k_s = 18$ smoothing neighbors on 1,000 selected genes for Visium HD and all 422 imported genes for Xenium, followed by a 30,000-location PCA/k-means fit and full-data projection and assignment. Bottom: neighborhood sizes, location counts, raw BD, expected raw BD, and relative BD for the three label maps. Expectations are analytic under random relabeling with fixed label counts; each row summarizes one map.

bioRxiv preprint doi: https://doi.org/10.64898/2026.10.26.761912; this version posted October 30, 2026. The copyright holder for this preprint (which was not certified by peer review) is the author/funder, who has granted bioRxiv a license to display the preprint in perpetuity. It is made available under a CC-BY 4.0 International license.

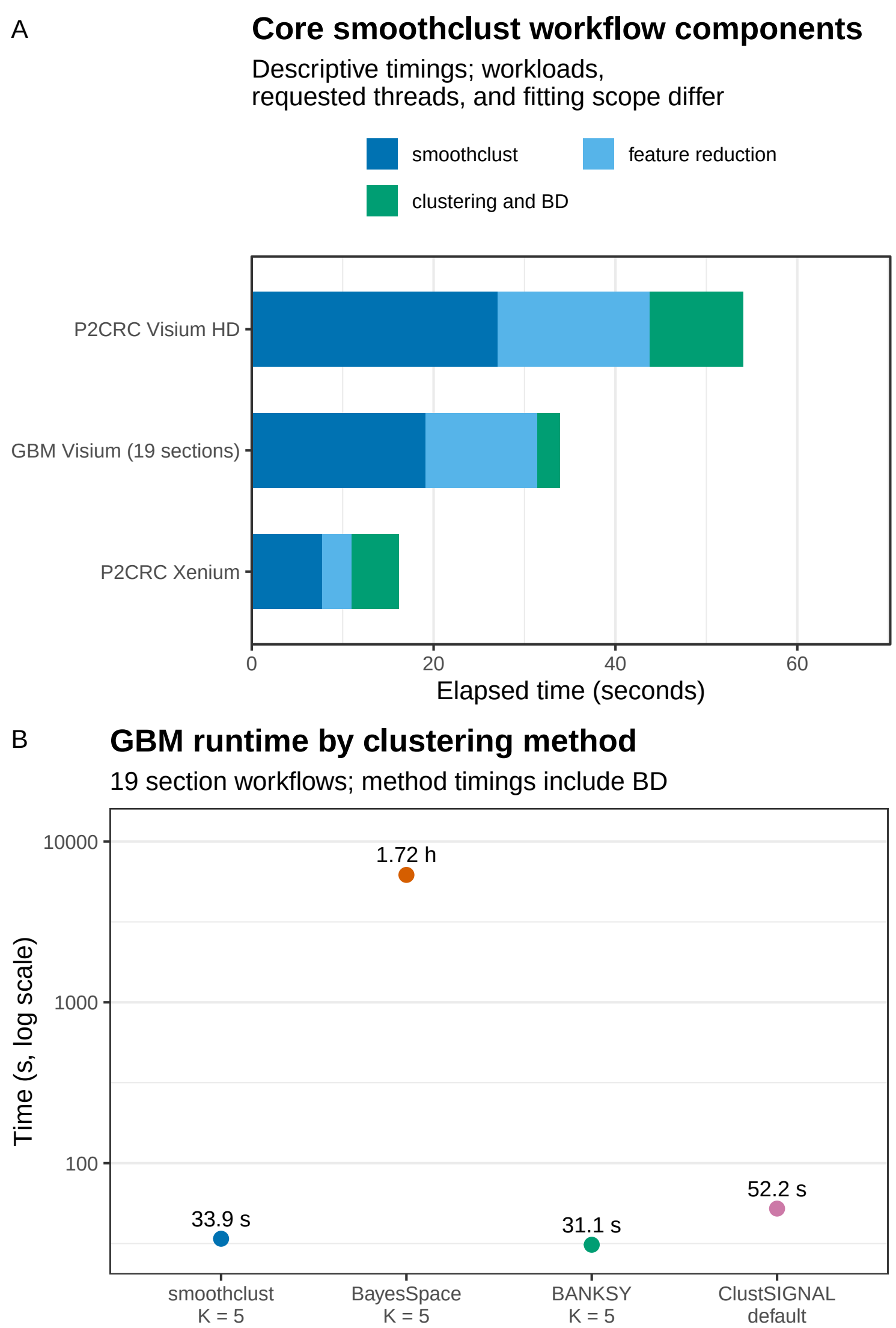

Supplementary Figure S16: Core smoothclust workflow components and GBM method-comparison runtimes. (A) Stacked timings for smoothing, feature reduction, and clustering plus BD in the GBM Visium, P2CRC Visium HD, and P2CRC Xenium analyses, on a linear axis. The GBM total sums per-section $K = 5$ component timers; Visium HD and Xenium are directly observed single runs. Workflow sizes, fitting scope, timing exclusions, requested nearest-neighbor threads, and hardware are given in Supplementary Table S2. (B) Runtime comparison for smoothclust, BayesSpace, BANKSY, and ClustSIGNAL on the same processed GBM/glioma data, on a logarithmic axis. The first three used $K = 5$; ClustSIGNAL used defaults. The smoothclust time reuses the component-derived GBM total; external-method totals sum per-section timers. Method-comparison runtimes include method-specific preprocessing and dimension reduction where applicable, clustering or labeling, and the BD metric calculation; they exclude download, import, shared filtering, output, and plotting. GBM requested 14 threads where supported.
